# Early exit: Estimating and explaining early exit from drug treatment

**DOI:** 10.1186/1477-7517-5-13

**Published:** 2008-04-25

**Authors:** Alex Stevens, Polly Radcliffe, Melony Sanders, Neil Hunt

**Affiliations:** 1 EISS, Keynes College, University of Kent, Canterbury, Kent CT2 7NP, UK; 2 The Institute for Criminal Policy Research, 8th floor, Melbourne House King's College London, Strand, London WC2R 2LS, UK; 3 KCA (UK), 44 East Street, Faversham, Kent ME13 8AT, UK

## Abstract

**Background:**

Early exit (drop-out) from drug treatment can mean that drug users do not derive the full benefits that treatment potentially offers. Additionally, it may mean that scarce treatment resources are used inefficiently. Understanding the factors that lead to early exit from treatment should enable services to operate more effectively and better reduce drug related harm. To date, few studies have focused on drop-out during the initial, engagement phase of treatment. This paper describes a mixed method study of early exit from English drug treatment services.

**Methods:**

Quantitative data (n = 2,624) was derived from three English drug action team areas; two metropolitan and one provincial. Hierarchical linear modelling (HLM) was used to investigate predictors of early-exit while controlling for differences between agencies. Qualitative interviews were conducted with 53 ex-clients and 16 members of staff from 10 agencies in these areas to explore their perspectives on early exit, its determinants and, how services could be improved.

**Results:**

Almost a quarter of the quantitative sample (24.5%) dropped out between assessment and 30 days in treatment. Predictors of early exit were: being younger; being homeless; and not being a current injector. Age and injection status were both consistently associated with exit between assessment and treatment entry. Those who were not in substitution treatment were significantly more likely to leave treatment at this stage. There were substantial variations between agencies, which point to the importance of system factors. Qualitative analysis identified several potential ways to improve services. Perceived problems included: opening hours; the service setting; under-utilisation of motivational enhancement techniques; lack of clarity about expectations; lengthy, repetitive assessment procedures; constrained treatment choices; low initial dosing of opioid substitution treatment; and the routine requirement of supervised consumption of methadone.

**Conclusion:**

Early exit diminishes the contribution that treatment may make to the reduction of drug related harm. This paper identifies characteristics of people most likely to drop out of treatment prematurely in English drug treatment services and highlights a range of possibilities for improving services.

## Background

Although opioid maintenance is the central component of those drug treatment programmes that have been most clearly shown to reduce drug-related harm, these are most effective when provided alongside psycho-social support [1,2]. In the UK, much of this support is collectively termed 'structured treatment' and typically includes two main modalities: structured counselling and day programmes. Treatment provision largely comprises community-based programmes; however, a minority of people also enter residential rehabilitation services. Drug problems are not limited to opiate users and, in Britain, frequently comprise poly-drug use, or may be dominated by stimulant use – notably cocaine/crack. Consequently, some people's treatment is focused exclusively on the psycho-social support components.

Since 1998, the UK has seen a big expansion in provision that has led to a 113% increase in the numbers of people being assessed for such structured drug treatment [3]. Increasing attention is now being given to ensuring that those who are assessed for treatment are retained long enough to benefit from it. The available research on retention has tended to look at the predictors of retention over several months, but reveals that a large proportion of those who drop out do so in the first few days and weeks of treatment. For example, in an earlier study of retention in an English region, 48% of treatment clients dropped out within the first six months of treatment, and predictors of this drop out were examined. However, 26% of those who dropped out did so before two weeks in treatment, although the predictors of this early exit were not examined [4].

To date, 'early exit' has received little attention. Although the outcome of some assessments might be a judgement that treatment is not needed it otherwise probably represents a waste of resources, because time and money that is invested in initial contacts and assessment is lost when people do not go on into treatment. There is some possibility that the assessment itself operates as a brief intervention by enabling people to take stock of their situation and receive advice or information that leads to action. But in general it represents a missed opportunity for individual drug users to access and receive the help that they may need in order to achieve their own aims, such as reduction or cessation of drug use and improving their health. It is clear that there is attrition at each stage of the process – between referral and assessment, assessment and treatment and within the first month of treatment – and that there is a need to look at different ways of maintaining clients in services at these points of contact.

The retention literature points towards a number of individual and system variables that may also influence early exit. Individual factors include ethnicity; employment status; co-morbidity of mental health problems; gender; age; problems with drugs other than opiates; and, previous treatment experience [5-9]. System factors include: referral from the criminal justice system; waiting times; levels of support and contact during waiting times; the extent to which services are welcoming and empathetic; the use of motivational enhancement approaches; and, the dose-adequacy and speed of titration of opioid substitution treatment [10-20]. This existing literature is not extensive and is derived from services provided in varied cultural contexts with differing treatment systems: variables that proved significant in one study are not consistently found to be so in other investigations. There is also some suggestion in the available research [21] that different factors may be associated with dropping out before and after treatment entry. This is important, as if these factors can be identified; it would enable agencies to focus efforts on the most vulnerable people at the most appropriate stage of their treatment journey with service enhancements that are most likely to increase their engagement and success in treatment. The strongest influences on retention that have so far been found are system variables rather than individual factors; with people attending the poorest performing services being 7.1 times as likely to drop out early as those attending the best, which suggests that important determinants of early exit may be amenable to change through service improvements.

This article describes a mixed-method study that examined this phenomenon of early exit from drug treatment. It aimed to estimate the rate of early exit, to identify those drug users who are most likely to exit early, to analyse why they do so, and to provide recommendations for reducing early exit in order to boost retention, effectiveness and the impact of drug treatment. This paper is based on a fuller report that was originally provided to the research funders (available as a PDF version supporting document from the Harm Reduction Journal website). The full report provides more detail of the background to the study, methodology and, in particular, the qualitative analysis.

## Methods

It was anticipated that different factors would be associated with dropping out before treatment started and dropping out in the first month of treatment, so two stages of early exit are defined. The first refers to people assessed at a drug service, but who do not enter this programme (referred to as Exit1). The second refers to people who enter treatment (i.e. attend a first treatment appointment), but leave early (measured as staying less than 30 days in treatment and referred to as Exit2).

### Quantitative methods

From the previous research in this area, we developed the following hypotheses for testing through multivariate analysis.

1. That transition from treatment offer to treatment entry is negatively associated with (a) being male, (b) being a primary stimulant user, (c) being a member of an ethnic minority, (d) being homeless, (e) longer waiting times, (f) being younger, (g) treatment modality (i.e. other than substitute prescribing) and (h) with being referred by the criminal justice system.

2. That transition from treatment entry to retention in treatment at one month is negatively associated with the same factors (a-h).

3. That transition from assessment to retention in treatment at one month (i.e. any early exit) is negatively associated with the same factors (a-h).

4. That different factors predict drop-out from assessment to treatment entry and drop-out in the first month of treatment.

Hypothesis 4 may seem to contradict hypotheses 2 and 3, as it would be contradicted if both hypotheses 2 and 3 were completely confirmed. It is phrased in the way it is in order that we could test whether the null hypothesis (i.e. that there was no difference in the variables influencing exit at each stage) could be rejected.

We primarily used data available from the National Drug Treatment Monitoring System (NDTMS) through the National Treatment Agency for Substance Misuse (NTA). This is a standardised data set used nationally. Intentions to use additional data available in case-file records held by a random sample of drug treatment agencies in the three Drug Action Team (DAT) areas on which we focused were largely unsuccessful due to difficulties in obtaining it (these are discussed in the full report: Additional file 1).

Drug treatment services in England and Wales are categorised in four tiers. Tier 3 includes non-residential structured treatment, including prescribing, structured counselling and day programmes. Tier 4 includes residential programmes. The dataset provided from the NDTMS by the NTA included people who were in Tier 3 or Tier 4 treatment during 2005/6. The dataset provided from the NDTMS included cases with triage (i.e. assessment), start and discharge dates up to 31^st ^March 2006. People were selected for inclusion in the analysis if:

• Their most recent triage was before February 2006 and after 1^st ^April 2005. The cut-off date was before the end of the period for which data was available in order to give enough time for all the people in the analysis to have either entered treatment or dropped out.

• They entered any tier 3 or 4 treatment except inpatient detoxification (for which the planned length is often less than 30 days).

• They contacted treatment agencies in the three sampled DAT areas.

• They were 18 or over.

This produced a dataset that included 2,624 people.

Three outcome variables were included as dependent variables in the analyses:

1. Exit1. Drop out between triage and treatment entry.

2. Exit2. Drop out within 30 days of starting treatment.

3. Exit3. Any drop out between triage and completing 30 days of treatment.

People were coded as "yes" (1) on Exit1 if their first treatment episode had a triage date, but no start date. They were coded "yes" on Exit2 if their first treatment episode had a start date and a discharge date within 30 days of it. They were coded "yes" on Exit3 if they were "yes" for either Exit1 or Exit2. Bivariate analysis was performed using SPSS 14. We anticipated that there may be systematic differences in services and recording practices between agencies, which we would not be able to measure (e.g. there was no alternative record available to test whether agencies were systematically delaying their reporting of drop-out). We therefore used hierarchical linear modelling in order to test the influence of variables at both agency and individual level, while controlling for variation between the agencies. This was performed using HLM 6.

### Qualitative methods

The aim of the qualitative research was to complement the quantitative analysis through an examination of the experiential, situational and attitudinal aspects of early exit. In order to examine these elements, a series of semi-structured qualitative interviews and a focus group was conducted in parallel with the quantitative analysis. Interview guides were developed with reference to the existing literature on early exit/engagement/retention in treatment, which was reviewed as part of the research process. The guides were structured to address distinct features of the treatment process through which people pass and included prompts that were used to examine issues that were not mentioned spontaneously. They addressed questions regarding a) the circumstances of people who exit early, b) their reasons for non-engagement and, c) perceptions of system changes that might have improved retention. We involved drug treatment staff and people who have dropped out early from drug treatment in these interviews. We include the interview guide for service users (Additional file 2).

We carried out qualitative interviews with 53 clients; 22 from services in the metropolitan areas and 31 from services in the provincial area. We also interviewed 16 members of service staff. We recruited both clients and staff via the main treatment services. Clients were asked by treatment staff at the point of assessment if they would like to be interviewed by researchers from the project, should they drop out before twelve weeks.

We were fully aware in advance that service users who disengage rapidly from treatment may be harder to engage in allied research and we tried to address this by the following methods:

• We paid interviewees for their time and contribution.

• Care was taken to ensure that all information about the study was easily understood by people with low literacy.

• Other than recruiting people through treatment services, we also used snowball sampling from interviewees to identify other potential respondents [22].

As far as possible, we included interviewees with characteristics to reflect our theoretical concerns including males and females, a full range of age groups, members of different ethnic groups, offenders, users of different drugs and people who have exited from services at different times; i.e. between assessment and treatment provision and within the first month of treatment.

Analysis of qualitative data should, ideally, proceed until data saturation is achieved i.e. interviews no longer generate new themes. However, this is incompatible with a project that, by necessity, has a finite budget and fixed timeline. A recent study has reported that data saturation was achieved after 12 cases, with most significant themes emerging within the first six cases [23]. We had intended to recruit at least 20 people from each of the sub-groups implied by the main theoretical concerns described and although this aim has not been met in every instance, we believe that we have enough data from each group to be confident about results. No new themes relating to gender, type of drug use, offending and mental health were identified in the latest analysed interviews with women, heroin, poly-drug and crack users, recent offenders and those with mental health problems respectively. We are less confident that we achieved data saturation on issues relating to ethnicity.

The analysis of the qualitative interviews was shaped by our knowledge of the existing literature, themes that had emerged in previous reports and our knowledge of the data. Consequently, our analytical approach is best described as adaptive coding [24].

Throughout this article, participants have been anonymised (Table 1).

**Table 1:** Qualitative sample characteristics

	N		N
Age range		Gender	
*19–25*	9	*Men*	39
*26–30*	11	*Women*	14
*31–35*	7	Ethnicity	
*36–40*	9	*White British*	40
*41–45*	11	*Mixed heritage*	5
*46–50*	5	*Black British*	4
*>50*	1	*Irish*	2
Primary drug used		*Asian British*	1
*Heroin*	22	*Traveller*	1
*Poly-drug use*	14	Recent offender	
*Crack*	11	*Yes*	28
*Cannabis*	4	*No/not reported*	25
*Amphetamine*	1	Psychiatric comorbidity	
*Prescription drugs*	1	*Yes*	18
		*No/not reported*	35

### Sample description

Table 2 shows the characteristics of the sample that was included in the analysis of monitoring data, which included clients whether they dropped out or not. In general, the sample is typical of the caseload of English drug treatment agencies, in that they were predominantly white, male opiate users in their late twenties and thirties. Values of the referral source variable were combined to create a variable for whether the person was referred through the criminal justice system (CJS). A combined variable was also created for whether the primary drug was a stimulant. The distribution of ages showed several outliers above the age of 56. These ages were transformed to 56 in order not to distort the other analyses with their extreme values. The distribution of days waiting between referral and treatment start were highly positively skewed, with many zero values, and so could not be transformed to normality for use in parametric tests. They were instead recoded to create a dichotomous variable with a score of zero indicating a short waiting time and a score of 1 indicating a long waiting time (with the split between short and long defined as the median of 13 days). Just over half the sample had their triage assessment recorded as on the same day that they were referred. For those who had to wait for triage, the average days waiting was 21 (standard deviation: 49.5). In analysis, a dichotomous variable indicating whether the person waited any days between referral and assessment was used.

**Table 2:** Sample characteristics at entry

		n			n
Mean age (standard deviation)	32.8 (8.7)	2,624	Mean days waiting: referral – start	23.6 (58.8)	2,169
Proportion male	68.2%	2,624	Proportion waited for triage	49.60%	2,624
Ethnicity		2,624	Modality entered		2,136
*White*	81.8%		*Prescribing*	37.8%	
*Black*	6.7%		*Structured counselling*	33.5%	
*Asian*	4.5%		*Day programme*	5.8%	
*Mixed*	3.4%		*Residential rehab*	3.3%	
*Other*	2.6%		*Other*	19.1%	
Referral source		2,624	Primary drug at entry		2,624
*Self*	48.7%		*Heroin*	52.2%	
*GP*	8.6%		*Crack*	12.9%	
*Other drug service*	7.2%		*Cannabis*	11.8%	
*Probation*	5.9%		*Cocaine*	8.9%	
*Arrest referral/DIP*	5.1%		*Methadone*	3.0%	
*CARAT/Prison*	5.0%		*Amphetamine*	2.9%	
*DTTO/DRR*	3.4%		*Anti-depressants*	1.5%	
*Psychiatry*	2.3%		*Benzodiazepines*	1.3%	
*Others*	13.8%		*Primary drug is a stimulant*	24.7%	
*Referred through criminal justice system*	19.2%		Is a current injector at entry	17.8%	2,624
Drug Action Team		2,624	No fixed abode at entry	10.1%	2,417
*Kent*	54.1%				
*Islington*	28.2%				
*Waltham Forest*	17.6%				

In addition to the variables needed to test the hypotheses listed above, a variable on whether the person reported that they were a current injector was used in order to provide a further test of the influence of the type of drug use on early exit.

## Quantitative Findings

Overall, 24.5% of the sample dropped out at the stages that have been defined as early exit for this study. The proportion of the sample that dropped out before starting treatment was 16.7%, compared to 7.8% who dropped out between starting treatment and staying in it for 30 days. This means that over two thirds of those who dropped out between assessment and 30 days in treatment did so before they entered treatment.

### Bivariate analysis

Table 3 lists the characteristics that have been hypothesised to be associated with early exit and shows that several turned out to display significant associations in bivariate analysis. For these tables (and the following multivariate analysis), people who dropped out before treatment entry were excluded from the analysis of Exit2 (drop out within 30 days of entering treatment).

**Table 3:** Bivariate associations with early exit

	n	Exit1 (before start)	Exit2 (within 30 days treatment)	Exit3 (any early exit)
Sex		**	n/s	**
*Male*	1,789	18.3%	9.8%	26.3%
*Female*	835	13.4%	8.4%	20.7%
Ethnicity		**	n/s	**
*White*	1,945	17.9%	9.9%	26.2%
*Other*	679	13.3%	6.9%	19.7%
Referral source		**	n/s	**
*CJS*	507	24.1%	7.5%	29.7%
*Other*	2,117	15.0%	9.7%	23.7%
Primary drug		*	n/s	**
*Stimulant*	657	19.9%	11.2%	29.8%
*Other*	1,967	15.7%	8.7%	23.2%
Injecting status		**	n/s	**
*Current injector*	466	9.4%	6.9%	15.7%
*Not current injector*	2,158	18.3%	9.9%	26.4%
Housing status		**	n/s	**
*Is NFA*	265	26.0%	12.2%	35.1%
*Not NFA*	2,143	14.8%	9.2%	22.7%
Wait for triage		n/s	n/s	**
*Any*	1,302	18.2%	10.6%	26.8%
*None*	1,322	15.3%	8.1%	22.2%
Wait for treatment			n/s	n/s
*Long*	1,106	-	9.5%	9.5%
*Short*	1,028	-	9.6%	9.6%
Type of service			**	**
*Prescribing*	806	-	6.7%	6.0%
*Other*	1,329	-	11.7%	11.7%

* p < 0.05, **p < 0.01

The average age of those who exited early was significantly lower than of those who continued in treatment at both stages of early exit. Those who dropped out before starting treatment had an average age of 31.9, compared to 32.9 for those who did not (p < 0.05). The difference for those who dropped out between entry and 30 days in treatment was 31.7 to 33 (p < 0.05). And the average age of those who dropped out at any stage between assessment and 30 days in treatment was 31.9, compared to 33 for those who stayed in treatment at this stage (p < 0.01).

In the cross-tabulation analyses presented in Table 3, the general pattern was that characteristics of the sample members were significantly associated with exit before treatment but not exit in the first 30 days of treatment for all the variables for which data on this earliest stage of exit was available. The wait from referral to starting treatment was not associated with early exit. It is interesting that referral through the CJS was associated with a greater likelihood of dropping out before treatment started, but a lower likelihood of dropping out within 30 days of starting treatment (although the difference was not significant at this stage, and not big enough to cancel out the effect of drop out before treatment on the overall rate of early exit).

The modalities that had the highest rates of drop out between entry and 30 days in treatment were day programmes, structured counselling and residential rehabs, at 15.4% 14.3%, 12.7% respectively.

### Agency differences

**Figure 1:**
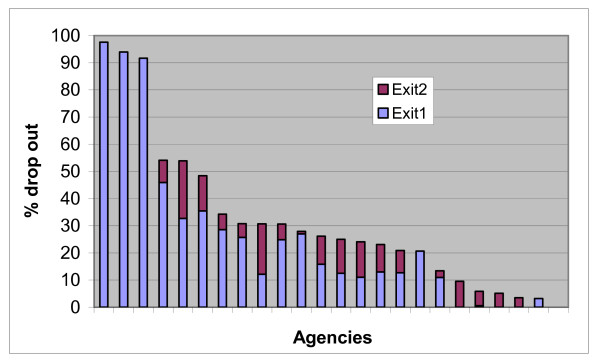
Rates of early exit by agency (includes only agencies with at least 20 people entering treatment).

As reported above, we anticipated that there would be differences between agencies in the rate of drop-out. Large differences in retention between agencies have been found by earlier studies [4,25], and these were also found in this study. Figure 1 shows the rates of early exit that appear in the monitoring data for those agencies that reported at least 20 people entering treatment in this dataset. Each bar in the chart represents a separate agency. There is a high degree of variability between agencies, with a range of between 97.6% and 0% dropping out at the early exit stages. The extremes of this range are represented by reasonably small agencies. The three with the highest and the two with the lowest rates of early exit reported less than 88% people entering treatment (over two thirds of people in the dataset entered treatment at larger agencies). Although disparities in retention rates have been found by other studies, it does not seem plausible that such large differences could arise without there being some differences in recording practices between agencies. For example, it is unlikely, given the relatively common occurrence of exit at both stages of early exit, that several agencies had no clients dropping out at either one of these stages. Yet this is what Figure 1 would suggest. It is very likely that some agencies have much lower rates of early exit than others, but this effect may be being exaggerated (or, in some cases, masked) by different recording practices for dates of entry and exit to and from treatment.

### Hierarchical linear modelling

The technique of hierarchical linear modelling (HLM) allows agency effects to be taken into account. Each of the significant variables in Table 3 was included at level 1 in separate HLM models with Exit1, Exit2 and Exit 3 as the outcome, dependent variable and with the agency that they contacted at level 2. Three characteristics of the agencies were also included at level 2 in separate HLM models; agency size (dichotomous around the median of 6 assessments in the sampled period), the agency's mean waiting time between referral and triage (dichotomous around the median of 6 days) and that agency's mean waiting time between referral and start of treatment (dichotomous around the median of 20 days). The variables that were significant in these separate models were then entered together into the final models. Of the agency characteristics, the size and mean wait for treatment were not significant in the separate models. Neither were the sex, referral source, primary stimulant use, and the waiting time for triage or treatment of the service users. These variables were therefore not included in the models reported in Table 4.

**Table 4:** HLM models of early exit

	Exit1 (before start)	Exit2 (within 30 days treatment)	Exit3 (any early exit)
Agency has high mean wait for triage		2.47**	
95% confidence interval		1.25 – 4.9	
Is of white ethnicity	1.28**		
95% confidence interval	(1.05 – 1.57)		
Has no fixed abode			1.37**
95% confidence interval			(1.1 – 1.71)
Is current injector	0.72*		0.68**
95% confidence interval	(0.59 – 0.88)		(0.56 – 0.82)
Treatment is prescription		0.37**	
95% confidence interval		(0.19 – 0.72)	
Age	0.88**	0.98**	0.87**
95% confidence interval	(0.81 – 0.97)	(0.97 – 0.998)	(0.8 – 0.96)

Population average models with robust standard errors

* p < 0.05, **p < 0.01

Blank cells indicate variable not included in final model

The odds ratios reported in this table show the predicted likelihood of a person exiting early, given the characteristics included in the models. They suggest that younger people were more likely to drop out early. For each unit increase in the standardised age variable (i.e. the standard deviation in age, or 8.7 years), the predicted odds of exiting early at any stage reduced by a factor of 0.87. In this analysis, CJS referral and being of no fixed abode were not predictive of exit before treatment entry (Exit1), but those who were of white ethnicity and those who were not current injectors were significantly more likely to drop out at this stage. Apart from age, no other personal characteristics were predictive of exit between treatment entry and 30 days (Exit2). Being in prescription treatment was strongly associated with retention at this stage. Younger age, not being a current injector and being of no fixed abode were significantly associated with any early exit (Exit3).

We were limited in the characteristics of agencies that were available to us in the data. Of the three that were present in the data (size, mean wait for triage and mean wait for treatment), only the mean wait for triage was significantly associated with one of the stages of exit. People who entered treatment at an agency that had a higher than median mean waiting time for triage were 2.47 times more likely to drop out before 30 days in treatment than were people who entered treatment at an agency with low mean waiting times for triage.

The HLM analysis supports the hypothesis that individual characteristics were significantly associated with early exit for some characteristics, but not others. One variable that was not included in the original hypotheses but was present in the dataset and in the analyses was also consistently predictive of any early exit in all three forms of analysis. People who reported being a current injector were less likely to exit early than those who did not. This mode of drug use seemed to be more influential than the actual type of drug consumed in influencing early exit.

These quantitative results suggest that homelessness, not being a current injector, being young, and being referred by the criminal justice service are important characteristics that are associated with early exit from treatment. The high variation in rates of early exit between agencies, the low rate of early exit from prescribing treatment and the finding in HLM that people are more likely to drop out in the first few days of treatment at agencies with high median waiting times for triage suggest that individuals' characteristics may not be as influential as the type (and quality) of service that is provided. The qualitative data enabled us to look at these characteristics of individuals and services in more depth.

## Qualitative findings

This part of the article describes findings from the qualitative interviews with service users and practitioners. These are much more comprehensively reported, including quotations from clients and staff in the full report which can be found as a PDF link on the Harm Reduction Journal website. Here we have restricted our description of the findings to a summary of the main themes that emerged.

### Explanations offered for early exit

As can be seen in Additional File 2, the service user interview guide proceeded through open questions about people's reasons for early exit in their own terms before probing of *a priori *factors that have previously been linked to disengagement/retention within the literature.

Explanations for early exit from services were diverse and did not all signify treatment failure. For example, some clients reported that they had dropped out of treatment because they had become abstinent and therefore that they not feel that treatment was needed. Other clients who were in contact with a range of health and social care services had disengaged because they felt they were receiving adequate support from elsewhere. Nevertheless, many of the explanations offered by clients for early exit point to matters that may be important for understanding how engagement might be improved.

### Motivation vs. Treatment Options as Explanations for Disengagement

A number of former clients primarily attributed their disengagement to their own motivation rather than any fault of the treatment agency. This very much reflects professional discourse that locates responsibility for drug misuse and disengagement with the individual alone. Both our data and the research literature however indicate that motivation can be developed or discouraged by the treatment agency [15,26] Thus if motivation is viewed as mutable and arising from the dynamic interplay between the person and the service – rather than simply as an intrinsic property of the person – it appears that service factors such as waiting times, cancelled appointments and protracted assessment processes can each operate to diminish motivation. Conversely, clients reported that letters and phone calls from the service after a missed appointment had encouraged them to re-engage.

Four clients interviewed had attended private clinics for prescriptions, detoxification and residential rehabilitation and one, who was unable to get buprenorphine through the NHS, was buying it through the internet. This suggests that for some drug users it is less a problem of motivation and more a question of poor correspondence between how or what treatment is offered and the person's perceived needs. The mismatch between need and what was offered arose most often in two areas: access to residential rehabilitation and the prescribed treatment that was offered.

Although service users were often aware of its expense, many felt that residential rehabilitation was the best hope they had of distancing themselves from both their dependence and the factors that triggered relapse; but of those who had requested residential rehabilitation few were able to obtain it, or had been considered for it.

Likewise, many clients who had an expressed preference for buprenorphine rather than methadone had been denied this option. Staff reports of the relative costliness of buprenorphine compared to methadone suggest that there may be a post-code lottery effect in substitute prescription, or that there are restrictive criteria for prescribing buprenorphine. Among those people receiving methadone, service users described problems with low initial doses and slow titration (i.e. these low initial doses were only gradually increased to levels that they were comfortable with), which meant that they were likely either to drop-out of treatment or to use street heroin 'on top'. While there may be sound clinical reasons for slow upwards titration of methadone, both this practice and clients' concerns over the availability of buprenorphine and of tier four (residential) services referrals, point, in our view, to the need for both the criteria for and the methodology of treatment to be made explicit to service users. Clients reported in addition that problems with prescribing were sometimes exacerbated by other factors including the attitude of pharmacy staff – most opioid substitution treatment is subject to supervised consumption in these settings – or a requirement to attend a pharmacy that was hard to get to.

### Service Factors for Disengagement

Most factors identified by service users and practitioners as affecting early exit appear, to some extent, to be under the control of drug treatment agencies e.g. bureaucracy, waiting times and the lack of treatment options. Yet, although practitioners were often aware of factors that deterred service users from remaining in treatment, many felt that resource constraints, organisational structures or bureaucracy imposed by senior management meant these were outside their control.

### The Drug Service Building

Views contrasted more regarding whether or not the setting of the service itself (specifically the state of repair and layout of the building) had a negative impact. Whereas workers felt this was important, few service users considered this to be an issue and some felt that the neglected state of the service was less intimidating. Indeed, the vast majority of service users were relatively positive about their first visit and mentioned that both the staff and the building gave a good first impression. However, of those who had largely positive first impressions, not all stayed that way, with several feeling that once they had been assessed, the good service came to an end.

### Staff

Service users made assorted negative comments about staff – usually their 'keyworker'. These included: a sense of being belittled; resentment at being treated by someone who they perceived to have little direct understanding or experience of drug problems; and comments on what was seen as a general detachment and unresponsiveness of staff who left them feeling that they had been offered a service that was available rather than one that they had either asked for or that met their needs. There were, nevertheless, contrasting accounts by service users of staff who were well-regarded and who they felt had made an important difference to their lives, and some service users explained the staff attitude in terms of under-resourcing and strains on the service. Unwieldy, repetitive assessment processes were identified by both staff and service users as one factor that contributed to this.

### Waiting Times

Consistent with the quantitative data, reported waiting times varied considerably between services and often seemed to exceed national targets. As expected, longer waiting times were often referred to by staff and service users as having an adverse effect on engagement. Other problems were reported by clients to have arisen once they had entered treatment. For those who had stopped using drugs, mixing with service users who were still using in day programmes for example was reported to be distracting and encouraging of relapse. There were several reports by service users of drugs being used within the day programme service. Allied problems were reported by younger clients who did not use opiates, yet were exposed to the dominant, older, opiate using group, who were often entrenched in the criminal justice system.

### Dislike of Counselling

Finally, some clients reported that they were deterred by the general therapeutic ethos of counselling and group-based work. Within counselling, the simple expectation of talking openly about drug problems with a stranger was a problem for some clients. And besides the problems described above, groups sometimes included people whom the service user already knew from within their social network, and with whom they did not wish to associate or disclose personal information.

### Drug Using Network

Services were perceived by practitioners and service users to be oriented towards older, male, opiate users, who did tend to comprise the larger part of the population using the services studied. In particular, women and younger people sometimes reported that they viewed services as being less well geared to their needs. There was also concern that by using the service, there was a risk of being misidentified as a 'smack head', as opposed to say someone who *just *had problems with cannabis – with the lesser degree of stigma this was perceived to carry. By contrast, there was no evidence from client interviews that services were seen as inaccessible to black and minority ethnic (BME) groups, which is consistent with service utilisation patterns that were in proportion to the general population. This is consistent with our quantitative finding that it was white people who were more likely to exit before treatment entry. However, our BME sample was not large and the interviewers were white, which may mean that problems here are not reflected within the data.

### Male-Domination of Services

Although our quantitative data suggested that gender was not a significant predictor of early exit, practitioners felt that women, particularly those referred by the criminal justice system, were *more *likely to be retained in treatment than their male counterparts, once engaged. Nevertheless, some clients who were parents of young children identified problems with childcare that impeded attendance. They also described reluctance to attend treatment services because of concerns that social services would be alerted to their drug use.

For female commercial sex workers in the provincial area there was the suggestion in both staff and client interviews that the nocturnal nature of the work meant that services that operated between nine a.m. and five p.m. were not so accessible. There were also indications from both staff and client interviews that the relatively dense social network of drug users could result in women having to use services that were simultaneously attended by male perpetrators of domestic violence – a particular problem within day programmes that involve a strong emphasis on group-based work.

### Challenging the Notion of Chaos

Practitioners often discussed a 'chaotic' sub-group of service users that were especially difficult to engage and retain in treatment; with crack cocaine use as a common feature and – to a lesser extent – benzodiazepines. In part, this group overlapped with commercial sex workers. However, on closer examination, the problems with such 'chaotic' clients seemed to be substantially explained by the relative impotence of psychosocial interventions for crack users – compared to opioid substitution plus psychosocial support. Again, there were indications from client and staff interviews that services were poorly geared to the requirements of people whose lifestyles were perceived to be more nocturnal; with endorsements for outreach, low threshold services that were more flexible and provided more assistance with basic needs, such as nutrition.

### Dual Diagnosis

Where clients had co-existing drug and mental health problems, the influences were diverse. Staff reported that severe problems sometimes meant that people were very determined to get help and engage well, but the accompanying disorganisation could also impede the process. Some clients who were involved with multiple agencies reported that they experienced intervention overload. Engagement was affected by the challenge of attending multiple appointments with multiple organisations who were not necessarily working as closely as they might. For these and other groups where a constellation of problems such as domestic worries, poor accommodation or financial concerns affected the person's ability to attend appointments, some practitioners described the particular importance of flexibility during the early phase of treatment, which they felt was beneficial.

### Criminal Justice Referrals

Irrespective of whether they had come to the service 'voluntarily' or via the criminal justice system, most respondents reported that the decision to seek treatment was largely their own, even though there was often pressure to seek help in the background from the family or social care agencies. In a minority of cases clients felt that attendance had been imposed by the criminal justice system and that their subsequent disengagement was a consequence of this.

### Poor Information Base

Many service users interviewed felt they did not know enough about what to expect of treatment and few felt adequately prepared. There was evidence from service user interviews that out-dated or unfounded word-of-mouth information about drug services could be influential in whether or not drug users sought treatment at all: common themes included long waiting times, difficulty in accessing treatment and lack of support. There was also evidence of word-of-mouth information about the properties of methadone and its relative effectiveness compared to buprenorphine for example. In our view there is thus a need for drug services to produce targeted advertising of the services that can be provided and to counter myths about the effects of substitute opioids. In addition, it is our view that treatment services need to ensure that the expectations of those entering treatment are in line with what is available.

## Discussion

This study included a mixture of quantitative and qualitative methods. In this section we discuss how the findings of these methods converge or diverge, as well as their limitations in answering the questions we have sought to address.

### Comparing methods

In general, our quantitative findings on the kinds of drug users who were most likely to drop out early (i.e. those who were young, homeless, or not injectors) link well with our qualitative data, which suggested that the sampled services tend to be focused on the needs of the people who could be described as the traditional client group of drug treatment services; opiate users in their late twenties and thirties. Other drug users, who have different needs and are involved in different social networks may perceive such services as intimidating and excluding. There is some rationale to this, given the history of English drug problems, which have long been associated with heroin use and, more recently, with the threats of HIV and Hepatitis C to injecting drug users. It is also true that drug treatment services may have, in the form of opiate substitution drugs, more to offer heroin injectors who wish to move to less dangerous forms of drug use. Nevertheless, as 59% of problematic drug users have recently been estimated to be users of crack [27] and attention shifts to the needs of younger drug users who fit the alcohol-cannabis-cocaine-ecstasy profile described by Parker [28], it is increasingly important that drug treatment agencies develop services that can attract and retain people outside the traditional client group.

For some homeless drug users, consistent attendance at drug service appointments may raise practical difficulties and represent less of a priority compared to overwhelming housing needs. Particularly in the provincial DAT region, the perception that services are primarily oriented to opiate using men in their late twenties and early thirties seemed to deter younger and non-opiate users, both male and female from engaging with services.

There were other themes on which the quantitative and qualitative analyses seemed to concur. One was the apparently greater likelihood of early exit among men, although, as discussed above, this may be due to the greater proportion of CJS referred clients among men than women. Our quantitative analysis suggested that people who were referred by the criminal justice system were more likely to drop out between assessment and treatment entry, but not between entry and 30 days in treatment. Our qualitative interviews with people who had been referred in this way supported the findings of other research [29] that being involved with the CJS does not necessarily mean that drug users have lower readiness to change than other treatment clients. The high rate of drop-out after assessment suggests that some criminal justice involved drug users may be being referred inappropriately to day programmes, when they have expressed no willingness or need to enter treatment. This was found, for example, in the recently published evaluation of the Restriction on Bail pilot [30]. It may also be the case that CJS involved clients need more intensive support to get them from assessment to treatment entry, as they are likely to be dealing with a number of problems including legal, medical and housing needs, as well as treatment needs. Services that have been made available to offenders, such as rapid entry to treatment, may therefore enhance their early retention.

Our two data sources both suggest that some agencies are better than others at getting the basics right; i.e. providing a positive, welcoming environment to which drug service users wish to return once they have first encountered it. Once these basics are addressed, more systematic use of motivational interviewing and other counselling enhancements may offer the prospect of further improvements in retention rates. Specific examples include node link mapping – a technique which involves the counsellor and client creating a diagram of thoughts, feelings and actions and how they are linked, which has been found to increase client engagement and retention in treatment [31] and contingency management – the use of prizes, vouchers and/or clinic privileges in order to reward and incentivise good progress in treatment, which has been found to improve retention and outcome [32].

Perhaps the most important area of concurrence between quantitative and qualitative methods was on the sheer scale of the problem. The quantitative analysis suggested that nearly a quarter of people who contact drug treatment services and are assessed in our sampled areas do not go on to last a month in treatment, with over two-thirds of this drop out occurring between assessment and treatment entry. This fitted with reports in staff interviews of the high frequency of clients not turning up, especially for the first appointment. It also fitted with our drug user interviewees reports of multiple, and often short periods in contact with treatment. If drug treatment services are to maximise the opportunities afforded by the impressive increase in the numbers of people who are in contact with drug treatment, they will have to find ways in which to ensure that this contact lasts long enough to have an effect on the health, offending and other problems of dependent drug users.

One possible solution to the perceived domination of an in-group of drug users and associated features (such as the presence at drug treatment centres of people who are known as former dealers or victimisers) which make a service less accessible for others, is for there to be diversification of service location, including outreach/peripatetic working and a broader range of drugs services available in GP surgeries and health centres. This would enable drug users to contact professionals who could help them without having to go to a location that is perceived as excluding and/or stigmatising and with less risk of coming across people whom they fear or who may act as triggers to relapse. Another possible solution to the needs of drug users who are perceived to be "chaotic" is to increase the availability of open-access services that are open during the hours that they are likely to be needed (i.e. at night, as well as daytime). Both potential solutions pose challenges to the traditional pattern of providing drug services from centralised locations during office hours. Workers and managers with whom we discussed these potential solutions recognised the need for them. But they warned of how difficult it may be to make such changes, which are likely to require to changes in both commissioning frameworks and staff working practices.

### Limitations

As in every study, there are a number of limitations that should be borne in mind when reading and using these results. They include the generalisability of the findings from relatively small samples to the wider population of drug users and treatment agencies, the potential for recording practices to affect the quantitative data and our failure to recruit a large group of members of ethnic minority drug users for qualitative analysis.

Although our sample of over 2,500 people entering drug treatment services is large enough to enable powerful statistical analyses of the influences on early exit, it is small compared to the 181,000 people who entered drug treatment services in 2005/6. It is important to note that the DAT areas that were sampled had lower than average 12 week retention rates. This means that the reported rate of early exit from these areas is likely to be higher than that for all DAT areas. Nevertheless, the rates reported here indicate the large potential for improving longer term retention and outcome by improving retention in the first few days and weeks of treatment.

The research design incorporated both provincial and metropolitan areas in order not to distort the analyses by excluding one or other type of area, but the sampled areas may also have features and patterns of drug treatment services that are not shared by other areas. This problem is more acute for the qualitative sample, which included only 53 service users from 10 agencies. Although we considered that we achieved data saturation on most theoretical stratifications of interest (except ethnicity) within this sample, the practices and experiences of clients and workers at these agencies may be different to those at other agencies. Large differences between agencies emerged from our analysis. We did test the influence of some agency variables, such as size and waiting times, but we did not gather structured data that allowed us to investigate ways in which other features – such as staffing levels or training – relate to agency performance. This is an important area for future research.

We relied on the NDTMS data in our quantitative analysis and were not able, as we had hoped, to triangulate these data with data held in agency casefiles. As in all secondary data analysis, we were therefore limited to using the fields that were originally recorded, some of which were subject to missing data. This means that we gained a less complete picture of the influences on early exit than we might have done had we been able to collect primary, quantitative data. There is a specific issue with this NDTMS data, which is that it is used by the NTA to manage the performance of Drug Action Teams and of individual treatment agencies against a target for the proportion of clients who are retained for at least 12 weeks. This provides an incentive for agencies to under-record the extent of drop-out from their services. This may have affected the quality of the data that we used; a concern that is heightened by the discrepancies between agencies that are visible in Figure 1 above. This problem would have been more acute if we had been examining retention at 12 weeks, rather than one month, as there is more temptation to exaggerate treatment duration for clients who nearly make the 12 week target than there is for those who drop out more than 8 weeks before attaining it. The NTA has put much effort into improving the validity of the NDTMS data in recent years. However, it is still possible that recording practices vary between agencies and between workers in the same agency. The use of HLM should control for any systematic differences between agencies, but it should still be recognised that the data we used is produced through a social process with its own pattern of distortions and is not an exact facsimile of reality. For these reasons, our findings should be considered as suggestive and not a definitive description of the pattern of early exit from English drug treatment agencies.

All feasible studies are limited in their scope, and all discover valuable questions that they are not able to answer. We have uncovered aspects of drug service users, and of treatment agencies that deserve further attention in attempts to reduce early exit from drug treatment. Such further examination should involve:

• Comparative research on the practices (including recording practices), staffing levels and caseloads of those agencies with high reported retention rates, compared to those with low retention rates.

• Longitudinal research that is able to follow drug users through several episodes of treatment, in order to understand the cumulative effects of various stages in the treatment journey.

• Ethnographic research among groups of drug users to understand the flow of information between drug users about drug services and the effects of diversity, stigma and fear (e.g. of other drug users and of losing children to local authority care) within drug using sub-cultures on the decision to enter and stay in treatment.

## Conclusion

From our analysis of the data, we offer the following conclusions on estimating, explaining and reducing early exit.

In the sampled drug treatment areas, in 2005/6, the proportion of people in contact with structured drug treatment clients who exited between assessment and 30 days in treatment was 24.5%. More than two thirds of this early exit occurred between assessment and entry into treatment. However, there were wide disparities in rates of early exit between treatment agencies. From our qualitative data, it is likely that some of this early exit represent the attempts of problematic drug users to 'go it alone' rather than engage in treatment, but it still seems that high rates of early exit represent a waste of resources and opportunities to change lives for the better.

The characteristics of service users that were consistently associated with being more likely to exit between assessment and 30 days in treatment were being younger, being homeless (of no fixed abode) and not being a current injector. Age and injection status were both consistently associated with exit between assessment and treatment entry. Age was the only personal characteristic to be consistently associated with exit between treatment entry and 30 days in treatment. Those who were not in substitution treatment were significantly more likely to leave treatment at this stage.

Several treatment staff that we interviewed focused on characteristics of service users, such as the "chaos" of their lives and their lack of motivation in explaining why they leave treatment early. However, the existing research, our data on the different levels of early exit between agencies and the reports of other staff and service users whom we interviewed suggest that there is much that services can do to enhance the rate or retention in the first few days and weeks.

Drug users who do not belong to the traditional client group of injecting heroin users in their late twenties and thirties may find traditional drug services, provided from central locations in often run-down buildings, off-putting. The opening hours of services may exclude many potential clients, especially those who work (including those who work in the sex industry), from being able to attend treatment.

Some staff reported that they do make use of recommended techniques for enhancing early retention, such as proactive, personalised contacting for appointments and motivational interviewing during treatment sessions. However, the use of such techniques was not widely reported by staff or service user interviewees.

Our interviews with drug users also suggested that, by failing to publicise their services and to clarify expectations in advance of contacting treatment, treatment agencies leave the main source of information that drug users have about treatment to be the user's own previous (often unsuccessful) episodes of treatment or the conventional wisdom on drug treatment that is circulated through networks of drug users. It seems that clients referred by the criminal justice system may be particularly under-informed of what to expect from drug treatment.

There are things that drug services do that seem to deter some drug users from engaging in treatment. These include requiring drug users to go through repeated, lengthy assessment processes and multiple appointments to actually get treatment, not providing the treatment (especially residential rehabilitation and buprenorphine prescriptions) that some of our interviewees had hoped to get, insisting on supervised consumption of methadone, starting methadone prescription at doses that may be too low to help the drug user and mixing drug users who are at different stages of their 'treatment journey' in the same groupwork sessions.

This article has estimated the rate of early exit from treatment, has identified some characteristics of drug users and services that are useful in explaining early exit and has made some recommendations for how services may be able to reduce the rate of early exit in order to increase the quality and effectiveness of drug treatment. It is open to challenge or support by further research on the same issues. We hope that it will prove useful to policy makers and practitioners in the field.

## Competing interests

Potential conflict of interest: Neil Hunt is employed by one of the agencies that provided interviewees. However, his input was mainly at the research design stage. He did not play a direct role in analysis of data. There are no other competing interests.

## Authors' contributions

The responsibilities for acquisition and analysis of the qualitative and quantitative data were separate but overlapping. AS conceived and coordinated the study, led its design, gathered quantitative data and led on its analysis. PR conducted and analysed qualitative interviews. MS conducted and analysed qualitative interviews and gathered quantitative data. NH assisted with designing the research and providing qualitative data. AS and PR had lead responsibility for integrating the qualitative and quantitative analyses. All authors read and approved the final manuscript.

## Supplementary Material

Additional file 1 Early exit final report. Click here for file

Additional file 2 Interview guide and information sheet. Click here for file
