# Supplementary material for: Early exit: Estimating and explaining early exit from drug treatment
Source: Harm Reduct J. 2008 Apr 25;5:13. doi: 10.1186/1477-7517-5-13 (PMC2391146; doi:10.1186/1477-7517-5-13)
Supplement: Additional file 1 — Early exit final report. [file 1477-7517-5-13-S1.pdf]

FINAL REPORT

Early Exit:
Estimating and explaining early exit from drug treatment

Alex Stevens[1]
Polly Radcliffe[1]
Melony Sanders[2]
Neil Hunt [1,3]
Paul Turnbull[2]
Tim McSweeney[2]

[1]EISS, University of Kent
[2]Institute for Criminal Policy Research, King's College London
[3]KCA UK

Report prepared for the Department of Health
Drug Misuse Research Initiative (ROUTES)

February 2007

CONTENTS

1. Executive Summary 3

2. Introduction..7

3. Policy relevance 7

4. Literature review 9

4.1 Service characteristics...9

4.2 Client characteristics...11

4.3 Summary 12

5. Aims and objectives...13

6. Research design and method..14

6.1 Quantitative methods 14

6.2 Qualitative methods 16

6.2.2 Analysis...18

7. Quantitative Findings 19

7.1 Sample description..19

7.2 Results 20

7.3 Testing the hypotheses 25

8. Qualitative findings 28

8.1 Characteristics of service users exiting early 28

8.2 Referral and beyond 33

8.3 Service users' reasons for disengagement...............................34

9. Discussion 43

9.1 Comparing methods...43

9.2 Limitations 44

9.3 Further research 45

10. Conclusions...47

10.1 Estimating early exit 47

10.2 Explaining early exit...47

10.3 Reducing early exit. 48

Acknowledgements 50

References 51

This report forms part of the Department of Health Drug Misuse Research Initiative (ROUTES). This work was led by the University of Kent, which received funding from the Department of Health. The views expressed in the publication are those of the authors and not necessarily those of the Department of Health. The report is published by the University of Kent, Canterbury.

1. Executive Summary

Aims and objectives

The aim of this project was to provide information that would be useful to policy makers and practitioners in improving services for problematic drug users.

The project had four objectives:

1. To provide an estimate of the rates of early exit from tier 3 and 4 services in two regions of England - one provincial and one metropolitan.
2. To identify the characteristics of those dependent drug users who are most likely to exit early.
3. To provide information on why drug users leave early.
4. To make recommendations on how rates of early exit can be reduced.

By early exit, we mean leaving treatment between assessment and 30 days of treatment. We examine two stages of early exit: between assessment and treatment entry; and between entry and 30 days in treatment.

Policy relevance

As the numbers in contact with structured treatment (i.e. treatment for drug dependence which follows assessment and a care plan) have increased, so attention is being given to improving the quality and effectiveness of drug treatment services. It is widely accepted that treatment should last for at least 12 weeks in order to optimise its benefits. However, existing research indicates that many drug users leave treatment in the first few days and weeks.

If information from this project can be used to reduce drop out in the early stages of drug treatment, then this is likely to help policy makers and practitioners to achieve retention targets and to improve the effectiveness of treatment.

Background

Existing research in this area has focused on longer term retention than the early exit (thirty days or less of treatment) studied here. It suggests that the following individual characteristics are associated with dropping out of drug treatment in England:

- Young age.
- Male gender.
- Primary use of stimulants.
- Referral from the criminal justice system.

Research on the effect of waiting time is less clear, with some evidence that it does not affect retention once treatment has started, but may be associated with higher rates of drop out between assessment and treatment entry.

However, both US and English research tends to suggest that it is the characteristics of services rather than of service users that are more important in terms of influencing retention over several months. Some staff and agencies are better than others at retaining clients.

This study tests whether these service user characteristics are also important in influencing early exit. It examines the differences between agencies in early exit and suggests why some agencies may have higher drop out rates than others.

Methodology

This study adopts a comparative approach using two sources of data:

1. Quantitative data from the National Drug Treatment Monitoring System from three Drug Action Team areas for 2005/6. This dataset includes over 2,500 people.
2. Qualitative data from 16 staff and 53 service users in these areas, supplemented by discussion with other staff and service users in meetings and a focus group.

The quantitative data provides information on service user characteristics which is analysed using bivariate and multivariate (logistic regression and hierarchical linear modelling) methods.

The qualitative data was analysed using the adaptive coding approach. This uses existing theory and knowledge to inform the development of new concepts from the data.

The different analyses are compared and contrasted in order to improve the reliability of our interpretation through within- and between-method triangulation.

Findings

In the quantitative data, we found that 24.5% of the sample exited between assessment and 30 days in treatment. Over two thirds of this drop out occurred between assessment and treatment entry.

The characteristics of service users which were consistently associated with a greater likelihood of early exit between assessment and 30 days in treatment were:

- Being younger.
- Being homeless (no fixed abode).
- Not being a current injector at assessment.

These characteristics are significantly associated with early exit, even when the influence of other characteristics and of differences between agencies are taken into account.

Apart from younger age, different characteristics were associated with exit between assessment and treatment entry (referral from the criminal justice system, not being a current injector) and exit between entry and 30 days in treatment (not being in substitute prescription treatment).

We also found that there were very wide differences in the rates of early exit at different agencies.

Injecting drug users in their late twenties and thirties who are seeking prescription treatment can be seen as the traditional client group for drug services. Our quantitative findings support our qualitative finding that drug users who do not belong to this traditional service user group often find drug services off-putting.

Our qualitative research also suggested that drug treatment staff often use the concept of the unmotivated, chaotic drug user when explaining why people leave early from drug treatment. We challenge this explanation, using data from service users and previous research. We suggest that the very notion of chaotic drug users can be challenged and that it refers to drug users whose work and patterns of activity do not coincide with the nine-to-five opening hours of many drug treatment services. Therefore we suggest that:

- All drug users may be able to engage with services if these services are adapted to their needs.
- Motivation is mutable and can be developed or damaged by the quality and type of treatment offered.

From our interviews, it seemed that recommended techniques for enhancing motivation and engagement, such as motivational interviewing and proactive, personalised outreach are not widely used in the areas we sampled.

Drug treatment tends to be offered during office hours at central locations which become associated with the traditional client group. Other types of drug user (e.g. those who are younger, cannabis and crack users and parents) may be reluctant to attend these locations. Those who work, including sex workers, may not be able to attend during these hours.

Drug treatment services can also contribute to early exit by not publicising their services and waiting times (and so leaving other drug users' conventional wisdom as the main source of information for people considering treatment entry) and by not providing the service that people have come into treatment to get (e.g. residential rehabilitation and buprenorphine prescription).

Waiting times were not associated with dropping out of treatment in our quantitative data, but several of our interviewees referred to long waiting times and bureaucratic assessment processes as deterring them from contacting and staying with treatment agencies.

Implications for Policy and Practice

Based on our data and on previous research, we make 12 recommendations for reducing early exit.

1. There should be greater diversity in locations and opening hours of treatment, in order to avoid excluding potential service users who have difficulties in attending at limited times and places. This could include more offering of services in GP surgeries, as well as assertive outreach services.

2. Many drug users have inaccurate information and beliefs about drug treatment. Treatment agencies should therefore make a greater effort to describe and publicise their services to potential clients.

3. Services should offer more flexibility in prescribing (e.g. wider availability of buprenorphine and of rapid entry to prescribing).

4. There should be further examination of the safety and retention rates associated with methadone tolerance testing.

5. The levels of stigmatisation and inconvenience associated with supervised consumption of methadone should be reduced by offering privacy and flexibility in location and time of consumption.

6. Lack of childcare provision, fear of being reported to social services and of children being taken into care still deters many parents from engaging in drug services. Services should therefore implement existing recommendations to encourage parents with young children to engage in treatment.

7. Waiting times should be reduced at those agencies which are still missing the targets. This could be done by increasing staff:client ratios and ensuring that sufficient pharmacy slots are available.

8. More use should be made of motivational interviewing techniques early in the treatment episode in order to enhance motivation and retention.

9. Assessment processes should be adapted so that they are consistent with the development rather than destruction of tenuous motivation. Users should not have to attend several interviews before accessing treatment.

10. Services should implement existing recommendations and use proactive, personalised outreach during the waiting time and in response to non-attendance.

11. Special efforts are required to engage crack users in treatment. These could include rapid intake into treatment by staff who are knowledgeable about crack, as well as services such as relaxation techniques, cognitive behavioural therapy, complementary therapies, longer opening hours and the provision of food and transport.

12. Services will be more likely to retain homeless people, who are highly vulnerable to dropping out early, if they can rapidly assist with housing, welfare benefits and GP registration.

We recognise that such treatment enhancements will cost time and money. Whether they are cost-effective in improving health and offending outcomes should be tested in practice. Currently, many problematic drug users are in contact with structured drug treatment, but are getting little benefit from it.

2. Introduction

Since 1998, there has been a 113% increase in the numbers of people being assessed for structured drug treatment (NTA, 2006). This represents an impressive effort to increase the impact of drug treatment in reducing the individual and social harms of dependent drug use. However, increasing attention is being given to ensuring that those who are assessed for treatment are retained long enough to benefit from it. The available research on retention has tended to look at the predictors of retention over several months, but has highlighted that a large proportion of those who drop out do so in the first few days and weeks of treatment. This report describes a study that examined this phenomenon of early exit from drug treatment. It aimed to estimate the rate of early exit, to identify those drug users who are most likely to exit early, to analyse why they do so, and to provide recommendations for reducing early exit in order to boost retention, effectiveness and impact of drug treatment.

The report begins with a description of why the study is relevant to policy, before describing its aims, methods, quantitative and qualitative findings. These findings are then discussed before conclusions are drawn in the final chapter.

3. Policy relevance

The target to double the numbers in treatment has been achieved. The other main purpose of policy on drug treatment is to increase its quality and effectiveness. One indicator of both quality and effectiveness is the rate of retention in treatment. A large proportion of dependent drug users who make contact with drug treatment agencies in tiers 3 and 4[1] do not go on to engage in treatment, even when they have been assessed and offered a place in a treatment programme. Others begin treatment, but drop out within a month, therefore not satisfying the National Treatment Agency for Substance Misuse (NTA) Models of Care guidelines, which recommend that services should aim to retain clients for twelve weeks of 'case management' in order to maximise the potential benefits of treatment.

Particularly high rates of early exit have been observed among people referred into drug treatment from the criminal justice system, and especially in groups other than opiate users in their late 20s and early 30s. This issue also affects people who approach drug treatment through other routes (e.g. self-referral, or referral from a GP), although the precise size and distribution of the problem is not known (Millar, Donmall, & Jones, 2004; NTA, 2004).

One concern about access to treatment has commonly been that waiting times are a strong disincentive to engaging drug users in treatment (see below). The Department of Health has already funded research to look at the needs of service users and the impact that waiting times have on retention; it appears that although reducing waiting times is associated with increased rates of entry into treatment, it does not necessarily improve rates of retention in treatment (Donmall, Watson, Millar, & Dunn, 2005; Strang, Best, Ridge, & Gossop, 2004). Although waiting times for treatment are falling, the problem of continuing early exit from treatment becomes, relatively, an increasingly serious obstacle to increasing the numbers of drug users who participate and succeed in drug treatment programmes. This has been recognised within the Drug Interventions Programme, which includes assertive outreach to drug using offenders who drop out within its case management model.

[1] Tier 3 includes non-residential structured treatment, including prescribing, daycare, structured counselling and day programmes. Tier 4 includes residential programmes.

Early exit represents a waste of resources, because time and money that is invested in initial contacts and assessment is lost when people do not go on into treatment. And it represents a missed opportunity for individual drug users to access and receive the help that they may need in order to achieve their own aims of reducing their drug use and improving their health. It is clear that there is attrition at each stage of the process, between referral and assessment, assessment and treatment and within the first month of treatment, that different factors may contribute to causing drop out at different stages and that there is a need to look at different ways of maintaining clients in services at these points of contact.

This report will contribute to the development of policy and practice in this area by improving the evidence-base on the scale and nature of the problem of early exit from drug treatment and by making recommendations on how to reduce it.

4. Literature review

In this section we examine published research studies that have addressed issues of engagement and retention in drug treatment services. These studies have sought both to identify the characteristics of service users and of services that are likely to lead clients to engage or disengage from treatment. This research identifies particular points in the 'treatment journey' (National Audit Office, 2004) from referral to discharge, that can be opportunities or barriers for engagement. They have not specifically focused, as this study does, on early exit from drug treatment. It seems from the available literature that the factors related to retention in treatment can be examined in two categories: service characteristics and client characteristics.

4.1 Service characteristics

4.1.1 Waiting lists

Waiting lists and their reduction in drug treatment services have in recent years been the object of NTA targets, based in part upon the hypothesis that long waiting lists act as a barrier to engagement. A number of studies have examined the impact of waiting lists on engagement and retention in structured treatment. Donmall et al (2005) found that waiting times for treatment did not necessarily predict uptake of treatment or retention in treatment at three and six months. The authors argue that the service response to clients on the waiting list has a greater influence on uptake and retention than waiting times in themselves. More consequential for retention than the length of the waiting list is whether and how services maintain contact with clients on their waiting lists. Services can provide a variety of support to such clients, including telephone and written contact, motivational interviewing, complementary therapies and interim prescribing (Ibid). This is supported by Fischer et al's recent study of user involvement. Two of the recommendations of their large scale interview-based study in Scotland and England were that referrers should *increase* their level of support to clients during the time they are waiting for treatment to start and that no client contact made with referrers should go unacknowledged during this period (Fischer, Jenkins, Bloor, Neale, & Berney, 2007)

In their study of waiting times, Strang et al (2004) carried out a clinical trial based on the treatment outcomes for 182 opiate users in one outpatient substitute prescribing service. Half the research subjects were allocated to an accelerated treatment group and were referred to treatment within two weeks. The other half were allocated to a control group and referred to treatment within the standard waiting time. The study examined the effects of waiting lists on engagement and retention and found, like Donmall et al, that shorter waits were not necessarily associated with higher levels of retention, with a slightly *lower* proportion of the accelerated research subjects retained in treatment at three monthly follow-ups compared to those in the standard treatment group. While reducing delay was associated with successful treatment entry it did not improve the rate of treatment retention, compared to a group who entered treatment after a standard waiting time. So it is important to note that, while those who wait for longer may be equally, or even more likely to remain in treatment once they eventually start it, there may be many drug users who drop off the waiting list before they start treatment

Waiting times are therefore not irrelevant to the engagement of clients. Strang et al found that there was a comparative lack of improvement in drug-using behaviour among those who did not go on to enter treatment, which they argue emphasises the need to engage clients in treatment as early as possible. They recommend that 'high risk' clients (e.g. frequent users of crack who were less likely to enter treatment) could be targeted for accelerated treatment in

order to improve the rate at which they enter and are retained in treatment. Like Donmall et al, Strang et al (2004) recommend that treatment services stay in touch with clients during the waiting period 'to ensure that the service continues to be seen as a meaningful resource and to help maintain tenuous motivation'.

4.1.2 Service organisation

Meier (2005) also found that the organisation of the service was key in a survey of residential rehabilitation services in England. This study examined the factors associated with better client retention in this resource-intensive treatment mode. Improved retention was associated with higher quality, more pleasant (and more costly) treatment experiences with fewer beds, higher rates of single room occupancy, higher ratios of staff to clients and between one and two hours per week of individual counselling per week. Meier argues that residential rehabilitation services can be structured to improve retention rates and that services must take responsibility for the retention outcomes they achieve.

These points can be extended to other treatment modalities. A number of American studies cited in the NTA retention guide (NTA, 2005) provide examples of practices that maximise re/engagement and retention, including personal letters and motivational phone calls to service users in order to remind them of appointments and offering alternative appointments for 'no shows'. In the first of his 'Manners Matter' series in which he reviews studies of strategies for retaining clients in treatment, Ashton argues that:

> 'Treating the patient as an individual, being welcoming, empathetic, understanding and demonstrating respect and active, persistent caring are among the trademarks of services that hang on to clients' (Ashton, 2004: 4)

4.1.3 Enhancing motivation

Motivation may be ambivalent, and clients may have mixtures of motivation for entering treatment (Information and Statistics Division, 2004; Miller, Rollnick, & Conforti, 2002; Stevens, Berto, Frick, Hunt, Kerschl, McSweeney et al., 2006). It is therefore important that workers are sufficiently skilled and have received the necessary training in techniques to maximise engagement in order to prevent early exit. Motivational Interviewing (MI) is one such technique, designed specifically to develop motivation. It involves directive, client-centred interviews, in which trained counsellors elicit change in the client's behaviour by exploring and resolving ambivalence. In a meta-analysis of 72 studies (14 of which were on drug abuse), Hettema et al (2005) found that, compared to control groups who did not receive MI, this approach acts early in treatment and improves retention, adherence and staff-perceived motivation. They also found that the effects of MI weaken over time, suggesting that MI is most effective in early stages of treatment in preventing the early exit on which our study is focused.

These studies all demonstrate that the quality of the treatment service can encourage motivation, thereby increasing engagement and retention of clients. This is an important finding in that it problematises the notions of the 'chaotic' and unmotivated client which is common in discussions of treatment drop-out. Whereas, as will be seen in the discussion of our data, drug treatment staff often equate non-compliance with resistance and/or chaos, existing research suggests that drug users do make decisions with some extent of rationality about treatment options based, for example, on the quality and type of treatment available and the attitude and welcome demonstrated by treatment and other front-line staff (National Audit Office, 2004).

Edmunds et al's (unpublished) qualitative study of an arrest referral pilot scheme in the UK also found that offenders' choices about whether or not to enter treatment following arrest referral were based on rational sets of decisions. The authors argue:

> 'We have found a complex picture where decisions not to seek treatment are shaped by considerations relating to the police, beliefs about treatment assessments [and] about the social costs of abandoning their drug-using lifestyles' (Edmunds et al: 56)

4.1.4 Prescribing practice

Treatment approaches that take drug users' concerns seriously are therefore also likely to be more successful in engaging and retaining clients. This is the case with substitute methadone prescription where, because of the risk of toxicity there is a tendency for doctors to prescribe doses that are lower than those recommended in UK national guidelines, leading users to top-up with heroin, methadone or benzodiazepine and/or drop-out of treatment altogether (Department of Health, Scottish Office, Department of Health Welsh Office, & Department of Health and Social Services Northern Ireland, 1999). This brings us to the issue of tolerance testing in methadone treatment. A recent article in the British Medical Journal (Bakker & Fazey, 2006) reported on a service which offers same day appointments for opiate users seeking a methadone prescription. Rather than starting with a low dose in order to titrate upwards, doctors consult patients as to which dose of methadone will, in their experience, 'hold them', i.e. constitute a maintenance dose. After taking a supervised dose of methadone at a nearby pharmacy, patients are tested for methadone toxicity in the safe environment of the health centre. The authors contend that patients' judgements of their dose requirement have proved accurate and have led to very high retention rates. They argue that this practice 'may improve treatment uptake even for highly tolerant or high risk addicts' (Ibid: 1057)

In response, Zador and Farrell (2006) have argued in a subsequent edition of the same journal that the practice of basing initial methadone dosage on patients' judgements is dangerous, and not one to be generally advocated. They argue the issues of induction into methadone prescription and optimum maintenance dose are separate and it is the latter that requires further attention. They also indicate the difficulty that large, busy drug treatment services as well as small, inexperienced practices may have in providing the kind of supervision that is necessary for the type of tolerance testing advocated by Bakker and Fazey.

4.2 Client characteristics

The available research on characteristics that are linked to retention is mostly American. It tends to suggest that younger people, men, people who are not white, stimulant users, the unemployed, those with low motivation and those with mental health problems are more likely not to be retained for three months or more (DeLeon & Jainchill, 1986; Joe, Simpson, & Broome, 1998, 1999; Maglione, Chao, & Anglin, 2000; Peters, Haas, & Murrin, 1999). There are some exceptions to this pattern. For example, Sayre et al (2002) found that women were more likely to drop out and that those with mental health problems stayed longer in treatment than others. On criminal justice referral, some studies have suggested that the coercion associated with being ordered into treatment by the courts can improve rates or retention (e.g. Hiller, Knight, Broome, & Simpson, 1998).

This US finding on criminal justice system involvement enhancing retention is contradicted by the available English evidence. Beynon et al (2006b; 2006a) examined the characteristics of those who dropped out from treatment in a retrospective, longitudinal cohort of drug users entering treatment in the North West of England. They found that younger drug users and those referred via the criminal justice system (CJS) were more likely to drop out. The age, previous experience of the drug user and whether or not they had been referred via the criminal justice system were also found to be consequential for dis/engagement in an analysis of a sample of 2,616 clients entering treatment over a six month period in 2001, also in the North West of England (Millar, Donmall, & Jones, 2004). The greatest rate of drop out occurred in the first few days and weeks after treatment entry. At six months, just under half of the sample group were no longer retained in treatment. Ethnicity, drug type, and whether

or not the client had already been using methadone when entering treatment were not associated with retention or completion.

However, the authors also found that there were startling differences in the retention of clients between agencies, and that:

> '• clients attending the worst performing service were 7.1 times more likely to drop out early than clients attending the best. This finding indicates that the most important factor affecting outcome was the treatment service attended by the client' (Ibid: 4).

4.3 Summary

The available research focuses on retention over longer periods than this study of early exit, but has revealed that the first few days and weeks are crucial in ensuring longer term retention. It indicates some client characteristics that are important in predicting retention and may therefore indicate those groups of clients who are most vulnerable to leaving treatment early. These include age, type of drug use, the wait for treatment, the route of entry into treatment (and particularly whether it was through the criminal justice system). However, the available evidence suggests that the greatest influence on whether or not clients are engaged and retained in treatment seems to have something to do with the quality of the treatment service.

There is some suggestion in the available research (e.g. Strang et al 2004) that different factors may be associated with dropping out before and after treatment entry. This is important, as if these factors can be identified, it would enable agencies to focus efforts on the most vulnerable people at the most appropriate stage of their treatment journey with service enhancements that are most likely to increase their engagement and success in treatment.

This literature review has highlighted the issues that any study on early exit should seek to examine. Some of them, for example the characteristics of clients that are associated with early exit, are accessible by quantitative research. Others, for example, the reasons that drug users and treatment staff give for early exit, are more appropriate for qualitative examination. This study used both approaches in order to provide a rounded picture of the phenomenon of early exit.

5. Aims and objectives

This report aims to provide useful information to policy makers and practitioners on how many people exit early from treatment, on the factors that influence early exit and how this can be reduced. The specific objectives of the project were:

- To provide an estimate of the rates of early exit from tier 3 and 4 services in two regions of England - one provincial and one metropolitan.
- To identify the characteristics of those dependent drug users who are most likely to exit early.
- To provide information on why drug users leave early.
- To make recommendations on how rates of early exit can be reduced.

We anticipated that different factors would be associated with dropping out before treatment started and dropping out in the first month of treatment, so we define two stages of early exit. The first refers to dependent drug users who are assessed at a tier 3 or 4 drug treatment programme, but who do not enter this programme (referred to as Exit1). The second refers to people who enter treatment (i.e. attend a first treatment appointment), but leave early (this will be measured as staying less than 30 days in treatment and be referred to as Exit2).

From the previous research in this area, we developed the following hypotheses for testing through quantitative analysis.

1. That transition from treatment offer to treatment entry is negatively associated with (a) being male, (b) being a primary stimulant user, (c) being a member of an ethnic minority, (d) being homeless, (e) longer waiting times, (f) being younger, (g) treatment modality (i.e. other than substitute prescribing) and (h) with being referred by the criminal justice system.
2. That transition from treatment entry to retention in treatment at one month is negatively associated with the same factors (a-h).
3. That transition from assessment to retention in treatment at one month (i.e. any early exit) is negatively associated with the same factors (a-h).
4. That different factors predict drop-out from assessment to treatment entry and drop-out in the first month of treatment.

The aim of the qualitative research was to complement the quantitative analysis through an examination of the experiential, situational and attitudinal aspects of early exit. In order to examine these elements, a series of semi-structured qualitative interviews and a focus group was conducted. These addressed questions regarding a) finer details of the characteristics of people who exit early, b) their reasons for non-engagement and, c) perceptions of system changes that might have improved retention. We involved drug treatment staff and people who have dropped out early from drug treatment in these interviews.

The insights gained from both the quantitative and qualitative analysis are discussed in this report and, alongside the existing evidence, inform the recommendations that we present on how to reduce early exit.

6. Research design and method

Using only one method, and only one data source to study any phenomenon leads to dangers that the method is unreliable, or the data unrepresentative. Limiting research to either quantitative *or* qualitative methods can produce findings that can be seen as hard but shallow, or deep but soft. To avoid these dangers, and to strengthen the validity of our findings, we adopted a comparative approach (Fielding & Fielding, 1986). We aimed to triangulate between methods (quantitative and qualitative) and within methods (using different data sources). Investigator triangulation, with the involvement of two teams of experienced researchers from different Universities was intended to increase the robustness of project findings and recommendations. These were also enhanced by directly involving drug service users in the research design and analysis. We involved members of The Alliance (an organisation of current and former users of drug services) in the design of the qualitative interview schedule and in the analysis of qualitative results. We also discussed draft findings with a group of current service users in the provincial research area.

6.1 Quantitative methods

6.1.1 Sampling

In order to triangulate between data sources within the quantitative analysis, we intended to use data from two sources: data available from the National Drug Treatment Monitoring System (NDTMS); and data available in casefile records held by a random sample of drug treatment agencies in the three DAT areas on which we focused.

Following data screening and exploratory analysis, we aimed to use data from both sources to identify:

- the rate of attrition at the two stage of early exit – between assessment and treatment entry (Exit1) and between treatment entry and 30 days in treatment (Exit2) – and overall early exit between assessment and 30 days in treatment (Exit3).
- the characteristics (e.g. treatment type, gender, age, ethnicity, main problem drug, previous offending, housing situation, employment, wait between assessment and treatment entry, legal status) that are associated with attrition at the stages of early exit.

NDTMS data does not include information on employment so we intended to use the casefile data to examine this characteristic. However, there were major difficulties in acquiring data from client casefiles. We randomly selected 12 agencies from those providing tier 3 or 4 treatment in our sampled DAT areas. The plan was for treatment staff to obtain informed consent for researchers to view clients' files and for project researchers to then view the files and collect information for analysis. Despite repeated meetings and reminders, it was not possible to persuade treatment staff to obtain consent from a meaningfully large proportion of the clients who were assessed. As an alternative, we agreed with 10 of the 12 sampled agencies that they would provide us anonymised data from their casefiles. In some cases, this was done by administrative staff filling in a spreadsheet that we provided. This provided data that was usable. In other cases, the data was provided in the form of the data held in the agency's own client database. In one of these cases (agency C in table 6 below), the data was inconsistent and not usable for analysis. Specifically, many of the referral dates were after the recorded assessment date, and so waiting times could not be calculated. More importantly for our purposes, analysis using the discharge date recorded by the agency generated average durations in treatment which were implausibly high, suggesting that the discharge date was not an accurate record of the end date of treatment and so early exit was underestimated. Such problems particularly affected the services sampled in the metropolitan area, and may be

associated with the phenomenon of research fatigue, which affects services as well as clients (see qualitative methods section below). They meant that it was not possible to provide a meaningful comparison between data from the NDTMS and the casefile data. So, in this report, we focus our attention on the NDTMS data.

The dataset provided from the NDTMS by the NTA included people who were in tier 3 or 4 treatment during 2005/6. It was recoded (using syntax also provided by the NTA) to create one separate case for each individual person (so analysis refers to people, not treatment entries). Different treatment modalities and entries that were within three weeks of each other counted as a continuous episode.

The dataset provided from the NDTMS included cases with triage, start and discharge dates up to 31st March 2006. People were selected for inclusion in the analysis if:

- Their most recent triage was before February 2006 and after 1st April 2005. The cut-off date was before the end of the period for which data was available in order to give enough time for all the people in the analysis to have either entered treatment or dropped out.
- They entered any tier 3 or 4 treatment except inpatient detoxification (for which the planned length is often less than 30 days).
- They contacted treatment agencies in the three sampled DAT areas.
- They were 18 or over.

This produced a dataset that included 2,624 people.

6.1.2 Analysis

Using this dataset, we report descriptive data on the rates of attrition for the whole sample and for the various sub-groups, with the necessary caution that these bald rates are likely to be influenced by differences between the groups (e.g. age, gender, ethnicity). We test the significance of any differences by using chi-squared in cross-tabulation and t test in comparison of means (depending on the variables and their distribution).

In order to isolate the influence of the various factors, we use (as planned in the proposal) binary logistic regression to provide odds ratios for relevant groups at each stage of the route into treatment. We provide 95% confidence intervals for these odds ratios in order to indicate how likely it is that they are generalisable (Tabachnick & Fidell, 2001). The advantage of this method is that it allows examination of the independent effects of variables that are entered into the model. The odds ratio that is produced for each variable controls for the confounding influence of the other variables. For example, if people referred by the CJS also tend to be younger than those referred through other sources, the influences of age and CJS referral can still be independently examined. Through comparing the size and significance of the odds ratios for each variable, we are able to make judgements on which factors are most important in predicting early exit.

In order to check the effect of the various characteristics of those who contacted services, we also carried out a multilevel analysis, using hierarchical linear modelling (HLM, Bryk & Raudenbush, 2002). The advantage of this approach over logistic regression is that it offers a sophisticated method for analysing the influence of the characteristics of the service user (known as level 1 variables) while taking into account both the other level 1 variables and the differences between the services which they contacted (level 2). HLM can also produce odds ratios which indicate the difference in the likelihood of exiting early for a person with certain characteristics compared to others.

Three outcome variables were included as dependent variables in the analyses:

1. Exit1. Drop out between triage and treatment entry.

2. Exit2. Drop out within 30 days of starting treatment.
3. Exit3. Any drop out between triage and completing 30 days of treatment.

People were coded as "yes" (1) on Exit1 if their first treatment episode had a triage date, but no start date. They were coded "yes" on Exit2 if their first treatment episode had a start date and a discharge date within 30 days of it. They were coded "yes" on Exit3 if they were "yes" for either Exit1 or Exit2.

The bivariate and logistic regression analysis was performed using SPSS 14. The hierarchical linear modelling was performed using HLM 6.

6.2 Qualitative methods

We carried out qualitative interviews with 53 clients; 22 from services in the metropolitan area and 31 from services in the provincial area. We also interviewed 16 members of service staff.

6.2.1 Sampling

We were fully aware in advance that service users who disengage rapidly from treatment may be harder to engage in allied research and we tried to address this by the following methods:

- We paid interviewees for their time and contribution. Based on experience, we paid £15 in the provincial area and £20 in the metropolitan area to acknowledge the demands of the research, without constituting an inducement so powerful that it renders informed choice questionable (Ritter, Fry, & Swan, 2003; Seddon, 2005).
- Care was taken to ensure that all information about the study was easily understood by people with low literacy. Materials did not have a complexity greater than the Flesch Kincaid reading score for grade 6 (equivalent to that for an average 11 year old child).
- Other than recruiting people through treatment services, we also used snowball sampling from interviewees to identify other potential respondents (Atkinson & Flint, 2001). Interviewees were asked to recommend friends or acquaintances to the researcher; and the researcher followed up with interviewees at a later date to check whether they had any recommendations. In order to boost the size of the sample from black and minority ethnic groups, we paid interviewees £5 for referrals to people from people from these groups who had exited early from treatment.

We had originally planned to complete 30 interviews with clients from 6 randomly sampled services in the metropolitan area and another 30 from 6 randomly sampled agencies in the provincial area. However, despite adhering closely to the methods above, it was not possible to complete 30 interviews in the metropolitan area. Firstly, all of the sampled services were already involved in collecting consent from clients to take part in other research projects, particularly the current Drug Treatment Outcome Research Study (DTORS), and found it difficult to remember to ask clients for their consent for researcher contact, although consent forms were supposed to be added to the assessment package, and so the number of clients who consented to be contacted was limited. This was despite frequent contact with services by the researcher, both in person and by telephone. Research fatigue in drug services is a common phenomenon; one that we have discovered while working on other projects as well as this, and it has often proved hard to persuade workers to cooperate, despite frequent contact and updates. Secondly, one service which should have been able to provide us with a large number of clients is affiliated to the local Mental Health Trust and required the researcher to have honorary staff status before beginning the research. This unfortunately took many months to be arranged; thus the consent gathering process did not begin until September, which restricted the number of relevant clients from that particular service.

Table 1: Qualitative sample characteristics

	N		N
Age range		Gender	
19-25	9	*Men*	39
26-30	11	*Women*	14
31-35	7	Ethnicity	
36-40	9	*White British*	40
41-45	11	*Mixed heritage*	5
46-50	5	*Black British*	4
>50	1	*Irish*	2
Primary drug used		*Asian British*	1
Heroin	22	*Traveller*	1
Poly-drug use	14	Recent offender	
Crack	11	*Yes*	28
Cannabis	4	*No/not reported*	25
Amphetamine	1	Psychiatric comorbidity	
Prescription drugs	1	*Yes*	18
		No/not reported	35

As far as possible, we have included groups from several main theoretical concerns (see table 1), including males and females, different age groups, members of different ethnic groups, offenders, users of different drugs and people who have exited from services at different times; i.e. between assessment and treatment provision and within the first month of treatment.

Analysis of qualitative data should, ideally, proceed until data saturation is achieved i.e. interviews no longer generate new themes. However, this is incompatible with a project that, by necessity, has a finite budget and fixed timeline. A recent study has reported that data saturation was achieved after 12 cases, with most significant themes emerging within the first six cases (Guest, Bunce, & Johnson, 2006). We had intended to recruit at least 20 people from each of the sub-groups implied by the main theoretical concerns described and although this aim has not been met in every instance, we believe that we have enough data from each group to be confident about results. No new themes relating to gender, type of drug use, offending and mental health were identified in the latest analysed interviews with women, heroin, poly-drug and crack users, recent offenders and those with mental health problems respectively. We are less confident that we achieved data saturation on issues relating to ethnicity.

We do recognise that because we were attempting to contact drug users during office hours by letter and by telephone, those who agreed to be interviewed may be underrepresentative of those drug users who are homeless, involved in the night time economy, particularly sex work, those with mental health problems or in custody[2]. Because drug users are a highly mobile group and because mobile phones are often sold, or stolen we often found that we did not have up-to-date contact information for potential informants. Where we had telephone numbers we made repeated attempts to contact potential informants by telephone and persistence often paid dividends. We have also attempted to triangulate the qualitative findings with findings from the quantitative research and believe that our findings are as representative as possible given the scope of the study.

In addition, we carried out interviews with at least one member of staff at each of the treatment sites that are sampled in the quantitative analysis. This has enabled us to test the analysis of quantitative data and interviews with drug users against the experience and

[2] In addition, as we note in the Limitations of this study, the fact that the research team were all of white British ethnic origin may have played a part in our difficulties in recruiting participants of other ethnicities and led to an under representation of drug users from minority ethnic communities in the qualitative study.

perceptions of people who work with them. We consider that we have achieved data saturation with these interviews, as no new themes emerged for the latest analysed interviews.

We also took the opportunity to carry out a participative focus group which took place at the conference 'Drugs, Alcohol and Criminal Justice: Addressing the Balance', which was held at the University of Warwick from 13th to 15th March 2006. It involved 12 professionals from English drug treatment agencies and used techniques adapted from Participatory Appraisal[3] (Chambers, 1994) to discuss and test themes that were emerging from our review of the literature and from early interviews with staff.

6.2.2 Analysis

The analysis of the qualitative interviews was shaped by our knowledge of the existing literature, themes that had emerged in previous reports and our knowledge of the data. Our analytical approach could therefore be described as adaptive coding (Layder, 1998). There were two stages to the coding process. First, a random selection of interview transcripts was used to identify broad themes in the data. Second, a further ten interview transcripts were randomly selected and used to check whether or not any other broad themes could be added and to divide the existing broad themes into sub-themes. Once we were satisfied that most of the themes and sub-themes had been identified, they were imported into the Nvivo data processing package, along with the interview transcripts and field notes for all qualitative interviews. The use of Nvivo to code documents enabled two researchers to create a collaborative coding scheme for the interview transcripts. This facilitated the analysis of the interviews and enabled us to ensure that bias on the part of individual researchers was kept to a minimum.

It should be noted that throughout the report, we have anonymised clients – the names used bear no relation to the client's real name.

[3] Participatory appraisal is a an approach that recognises the people who directly experience a situation as experts on it and enables them to produce information about it using a range of graphical tools, including spider diagrams, timelines, maps, matrices and priority rankings.

7. Quantitative Findings

7.1 Sample description

Table 2 shows the characteristics of the sample that was included in the analysis of monitoring data. In general, they could be described as fairly typical of the caseload of English drug treatment agencies, in that they were predominantly white, male opiate users in their late twenties and thirties.

Table 2: Sample characteristics at entry

		n			n
Mean age (standard deviation)	32.8 (8.7)	2,624	Mean days waiting: referral - start	23.6 (58.8)	2,169
Proportion male	68.2%	2,624	Proportion waited for triage	49.60%	2,624
Ethnicity		2,624	Modality entered		2,136
White	81.8%		*Prescribing*	37.8%	
Black	6.7%		*Structured counselling*	33.5%	
Asian	4.5%		*Day programme*	5.8%	
Mixed	3.4%		*Residential rehab*	3.3%	
Other	2.6%		*Other*	19.1%	
Referral source		2,624	Primary drug at entry		2,624
Self	48.7%		*Heroin*	52.2%	
GP	8.6%		*Crack*	12.9%	
Other drug service	7.2%		*Cannabis*	11.8%	
Probation	5.9%		*Cocaine*	8.9%	
Arrest referral/DIP	5.1%		*Methadone*	3.0%	
CARAT/Prison	5.0%		*Amphetamine*	2.9%	
DTTO/DRR	3.4%		*Anti-depressants*	1.5%	
Psychiatry	2.3%		*Benzodiazepines*	1.3%	
Others	13.8%		*Primary drug is a stimulant*	24.7%	
Referred through criminal justice system	19.2%		Is a current injector at entry	17.8%	2,624
Drug Action Team		2,624	No fixed abode at entry	10.1%	2,417
Kent	54.1%				
Islington	28.2%				
Waltham Forest	17.6%				

Values of the referral source variable were combined to create a variable for whether the person was referred through the criminal justice system (CJS). A combined variable was also created for whether the primary drug was a stimulant. The distribution of ages showed several outliers above the age of 56. These ages were transformed to 56 in order not to distort the other analyses with their extreme values. The distribution of days waiting between referral and treatment start were highly positively skewed, with many zero values, and so could not be transformed to normality for use in parametric tests. They were instead recoded to create a dichotomous variable with a score of zero indicating a short waiting time and a score of 1 indicating a long waiting time (with the split between short and long defined as the median of 13 days). Just over half the sample had their triage assessment recorded as on the same day that they were referred. For those who had to wait for triage, the average days waiting was 21 (standard deviation: 49.5). In analysis, a dichotomous variable indicating whether the person waited any days between referral and triage was used.

In addition to the variables needed to test the hypotheses listed in section 4, a variable on whether the person reported that they were a current injector was used in order to provide a further test of the influence of the type of drug use on early exit.

7.2 Results

Table 3 shows the proportion of people who dropped out of treatment at the stages of early exit.

Table 3: Early Exit by DAT area				
DAT	n	Exit1 (before start)	Exit2 (within 30 days treatment)	Exit3 (any early exit)
Provincial	1,421	18.1%	8.7%	26.8%
Metropolitan1	741	11.9%	8.1%	20.0%
Metropolitan2	462	20.3%	4.3%	24.7%
**Total**	2,624	**16.7%**	**7.8%**	**24.5%**

Overall, nearly a quarter of the sample dropped out at the stages that have been defined as early exit for this study. Over two thirds of those who dropped out between assessment and 30 days in treatment did so before they entered treatment.

7.2.1 Bivariate analysis

Several of the characteristics that have been hypothesised to be associated with early exit turned out to display such significant associations in bivariate analysis, as seen in tables 4 and 5. For these tables (and the following multivariate analysis), people who dropped out before treatment entry were excluded from the analysis of Exit2 (drop out within 30 days of entering treatment).

Table 4: Age by category of exit			
	Exit1 (before start)	Exit2 (within 30 days treatment)	Exit3 (any early exit)
Significance	*	*	**
Mean age of drop-outs	31.9	31.7	31.9
Mean age of others	32.9	33	33

* p<0.05, **p<0.01

The average age of those who exited early was significantly lower than those who continued in treatment at both stages of early exit.

In the cross-tabulation analyses presented in table 5, the general pattern was that characteristics of the sample members were significantly associated with exit before treatment but not exit in the first 30 days of treatment for all the variables for which data on this earliest stage of exit was available[4]. The wait from referral to starting treatment was not associated with early exit. It is interesting that referral through the CJS was associated with a greater likelihood of dropping out before treatment started, but a lower likelihood of dropping out within 30 days of starting treatment (although the difference was not significant at this stage, and not big enough to cancel out the effect of drop out before treatment on the overall rate of early exit).

The modalities that had the highest rates of drop out between entry and 30 days in treatment were day programmes, structured counselling and residential rehabs, at 15.4% 14.3%, 12.7% respectively.

[4] It was not available for waiting time or type of treatment, and these variables were only completed if the person started treatment.

Table 5: Bivariate associations with early exit

	n	Exit1 (before start)	Exit2 (within 30 days treatment)	Exit3 (any early exit)
Sex		**	n/s	**
Male	1,789	18.3%	9.8%	26.3%
Female	835	13.4%	8.4%	20.7%
Ethnicity		**	n/s	**
White	1,945	17.9%	9.9%	26.2%
Other	679	13.3%	6.9%	19.7%
Referall source		**	n/s	**
CJS	507	24.1%	7.5%	29.7%
Other	2,117	15.0%	9.7%	23.7%
Primary drug		*	n/s	**
Stimulant	657	19.9%	11.2%	29.8%
Other	1,967	15.7%	8.7%	23.2%
Injecting status		**	n/s	**
Current injector	466	9.4%	6.9%	15.7%
Not current injector	2,158	18.3%	9.9%	26.4%
Housing status		**	n/s	**
Is NFA	265	26.0%	12.2%	35.1%
Not NFA	2,143	14.8%	9.2%	22.7%
Wait for triage		n/s	n/s	**
Any	1,302	18.2%	10.6%	26.8%
None	1,322	15.3%	8.1%	22.2%
Wait for treatment			n/s	n/s
Long	1,106	-	9.5%	9.5%
Short	1,028	-	9.6%	9.6%
Type of service			**	**
Prescribing	806	-	6.7%	6.0%
Other	1,329	-	11.7%	11.7%

* p<0.05, **p<0.01

Figure 1 shows the rates of early exit that appear in the monitoring data for those agencies that reported at least 20 people entering treatment in this dataset. Each bar in the chart represents a separate agency. There is a high degree of variability between agencies, with a range of between 97.6% and 0% dropping out at the early exit stages. The extremes of this range are represented by reasonably small agencies. The three with the highest and the two with the lowest rates of early exit reported less than 88 people entering treatment (over two thirds of people in the dataset entered treatment at larger agencies). It does not seem plausible that these wide disparities could arise without there being some differences in recording practices between agencies. For example, it is unlikely, given the relatively common occurrence of exit at both stages of early exit, that several agencies had no clients dropping out at either one of these stages. Yet this is what figure 1 would suggest. It is very likely that some agencies have much lower rates of early exit than others, but this effect may be being exaggerated (or, in some cases, masked) by different recording practices for dates of entry and exit to and from treatment.

Figure 1: Rates of early exit by agency (includes only agencies with at least 20 people entering treatment).

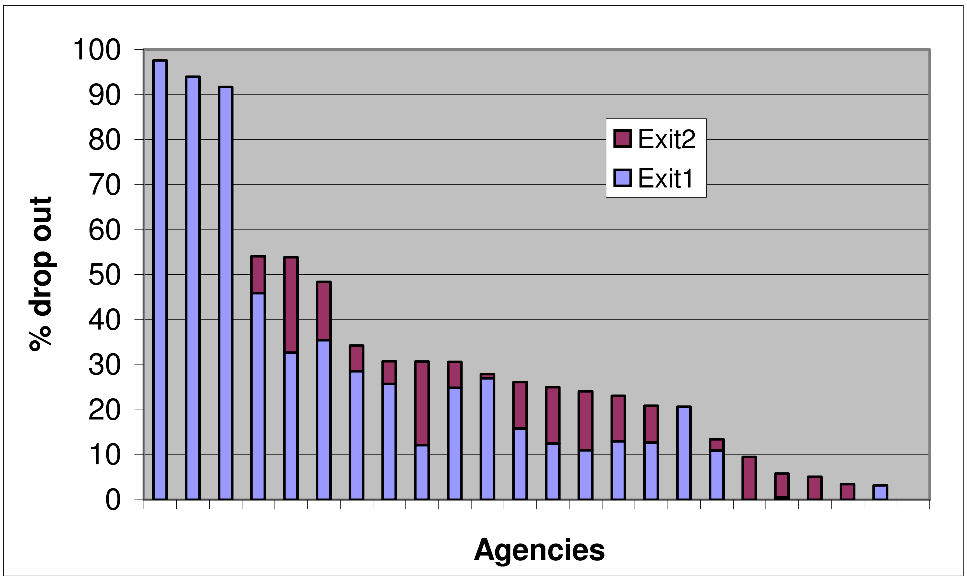

For further analysis, a variable was created that indicated the rate of overall early exit (Exit3) at the treatment centre that the person contacted (in five ordinal groups with approximately normal distribution, from low to high early exit rates).

Casefile data

The strong possibility that different recording practices affect the reported rates of early exit is supported by our analysis of the anonymised data that we gathered from the casefiles held by agencies in the three DAT areas where we did the research. Table 7 uses a dataset which includes adults at 9 of the sampled agencies who had a referral or assessment date at these agencies between 1st April 2005 and 31st March 2006.

The same outcome variables, Exit1, Exit2 and Exit3 were used as in the analysis of NDTMS data. Their frequency is shown in table 7.

Table 7: Early Exit by treatment site (casefile data)

Treatment site	N	Exit1 (before start)	Exit2 (within 30 days treatment)	Exit3 (any early exit)
A	13	0.0%	76.9%	76.9%
B	121	6.6%	65.3%	71.9%
C	299	40.5%	0.0%	40.5%
D	144	11.8%	18.1%	29.9%
E	105	3.8%	32.4%	36.2%
F	407	13.8%	21.6%	35.4%
G	113	2.7%	8.9%	11.6%
H	5	60.0%	0.0%	60.0%
I	53	1.9%	22.7%	24.6%
**Total**	**1,260**	**17.2%**	**20.3%**	**37.5%**

It again seems implausible that this distribution would arise without the influence of different recording practices. For example, the casefile data from agency C, a large prescribing agency

suggested that none of its clients dropped out in the first month of treatment. It also indicated an average duration of treatment, from first session to discharge, of 194 days (compared to a mean for all 10 agencies of 81 days). It is possible that the casefile records provided to researchers used the day the file was closed as the discharge date, and not the last day that the person was actually in treatment (as required by NDTMS guidance).

7.2.2 Multivariate analysis

Logistic regression

In order to test the effect of those variables that were significantly associated with any early exit in bivariate analysis, they were entered into logistic regression models with the three categories of early exit as the dependent variables[5,6]. People with missing values on any of these variables were excluded from the models. It was not possible to include the treatment type variable in the model for Exit3 (any early exit between triage and 30 days in treatment), as data was missing on modality for all those who did not enter treatment. The results are displayed in table 6.

Table 6: Multivariate associations with early exit (odds ratios)

	Exit1 (before start)	Exit2 (within 30 days treatment)	Exit3 (any early exit)
n	2,408	1,971	2,408
Nagelkerke R Square	0.063	0.035	0.054
CJS referral	1.65**	0.68	1.22
95% confidence interval	(1.28 - 2.12)	(0.45 - 1.04)	(0.97 - 1.53)
Has no fixed abode	2.03**		1.91**
95% confidence interval	(1.49 - 2.77)		(1.44 - 2.52)
Is white	1.63**		1.47**
95% confidence interval	(1.21 - 2.18)		(1.15 - 1.88)
Waited for triage	1.48**	1.41*	1.5**
95% confidence interval	(1.18 - 1.85)	(1.04 - 1.91)	(1.23 - 1.82)
Is current injector	0.44**		0.51**
95% confidence interval	(0.31 - 0.63)		(0.38 - 0.67)
Treatment is prescription		0.48**	
95% confidence interval		(0.34 - 0.68)	
Age		0.98	0.99
95% confidence interval		(0.97 - 1)	(0.98 - 1)

* p<0.05, **p<0.01
Blank cells indicate variable not included in final model

Prior to running these models, the data was tested for multicollinearity[7]. None was found. The strongest correlation between variables included in the initial models was -0.384 (p<0.01) between whether the person was a primary stimulant user and whether they entered prescription treatment. It should be noted that the values of Nagelkerke R Square[8] for these

---

5 Logistic regression is a technique which enables the calculation of the statistical association between characteristics (variables) of the service users and the likelihood of exiting early. The advantage of logistic regression over bivariate tests is that it enables these likelihoods to be calculated, while taking into account the effects of the other variables. So, for example, we can calculate the predicted likelihood of an older person exiting early, while holding constant the effect of their ethnicity and the other included variables.

6 Variables were entered backward stepwise in order to exclude variables that did not contribute significantly to the predictive power of the model. These variables thus excluded from all three models were sex, primary drug use and wait for treatment.

7 Multicollinearity indicates a strong relationship between the potential predicting variables. Its absence is an assumption of a robust logistic regression model.

8 Nagelkerke R Square provides an approximate measure of the proportion of variance in whether people exited early that is explained by the variables included in the model. It has a minimum value of 0 and a maximum of 1.

models are very small. This suggests that other influences, which were not included in the model (probably including agency effects) are important in predicting early exit.

Table 6 shows the odds ratios for the variables included in the final models. The odds ratio provides a measure of the likelihood (expressed in odds) that a person in that category of the included variable dropped out. People of no fixed abode were 1.91 times more likely to drop out early than those who had a different housing status. And people who reported current injecting status at triage were 0.51 times as likely as others to drop out early.

When controlling for the effect of other variables, the sex, primary drug used and waiting for triage were no longer significantly predictive of either of the stages of early exit. Different variables were again associated with different stages of early exit. Referral from the CJS, being of white ethnicity and having no fixed abode were significantly predictive of dropping out before treatment entry. Those who reported that they were currently injecting at triage were less likely to drop out at this stage. At the next stage of drop out, between entry and 30 days in treatment, none of individuals' characteristics were significantly predictive of exit. If the person had to wait for triage, they were more likely to drop out at this stage, but those who entered prescription treatment were less than half as likely to drop out as those who entered other modalities.

The strongest predictor in this analysis of any drop out between triage and 30 days in treatment was homelessness. People who reported that they were of no fixed abode were 1.91 times more likely than those who did not to fail to get to 30 days in treatment. However, it should be noted that this analysis does not incorporate the effect of the treatment centre at which the person was assessed. As shown in figure 1, rates of early exit vary widely between agencies. The technique of hierarchical linear modelling (HLM) allows these agency effects to be taken into account.

Hierarchical linear modelling

In order to provide a more sophisticated test of the influence of client characteristics on early exit, while taking into account differences between agencies (including recording practices) we carried out HLM analysis. Each of the significant variables in table 5 was included at level 1 in separate HLM models[9] with Exit1, Exit2 and Exit 3 as the outcome, dependent variable and with the agency that they contacted at level 2. Three characteristics of the agencies were also included at level 2 in separate HLM models; agency size (dichotomous around the median of 6 assessments in the sampled period), the agency's mean waiting time between referral and triage (dichotomous around the median of 6 days) and that agency's mean waiting time between referral and start of treatment (dichotomous around the median of 20 days). The variables that were significant in these separate models were then entered together into the final models. Of the agency characteristics, the size and mean wait for treatment were not significant in the separate models. Neither were the sex, referral source, primary stimulant use, and the waiting time for triage or treatment of the service users. These variables were therefore not included in the models reported in table 8.

Again, the odds ratios reported in this table show the predicted likelihood of a person exiting early, given the characteristics included in the models. The main difference between the results of the logistic regression and the HLM models is that, in HLM, age emerged as a significant predictor of both stages of early exit. Younger people were more likely to drop out early. For each unit increase in the standardised age variable (i.e. the standard deviation in age, or 8.7 years), the predicted odds of exiting early at any stage reduced by a factor of 0.87. In this analysis, CJS referral and being of no fixed abode were no longer predictive of exit before treatment entry (Exit1), but those who were of white ethnicity and those who were not

[9] For these models, age was standardised before entry.

current injectors were significantly more likely to drop out at this stage. Apart from age, no other personal characteristics were predictive of exit between treatment entry and 30 days (Exit2). Being in prescription treatment was even more strongly associated with retention at this stage in the HLM analysis than it was in the logistic regression. The same three variables – younger age, not being a current injector and being of no fixed abode - that were significantly associated with any early exit (Exit3) in logistic regression were also the significant variables included in the final HLM model for this stage.

We were limited in the characteristics of agencies that were available to us in the data. Of the three that were present in the data (size, mean wait for triage and mean wait for treatment), only the mean wait for triage was significantly associated with one of the stages of exit. People who entered treatment at an agency that had a higher than median mean waiting time for triage were 2.47 times more likely to drop out before 30 days in treatment than were people who entered treatment at an agency with low mean waiting times for triage. This suggests that a quick response to drug users when they present for a service may assist their early retention, although the effect was not large enough to be significant in the analysis of any early exit between triage and 30 days in treatment.

Table 8: HLM models of early exit

	Exit1 (before start)	Exit2 (within 30 days treatment)	Exit3 (any early exit)
Agency has high mean wait for triage		2.47**	
95% confidence interval		1.25 - 4.9	
Is of white ethnicity	1.28**		
95% confidence interval	(1.05 - 1.57)		
Has no fixed abode			1.37**
95% confidence interval			(1.1 - 1.71)
Is current injector	0.72*		0.68**
95% confidence interval	(0.59 - 0.88)		(0.56 - 0.82)
Treatment is prescription		0.37**	
95% confidence interval		(0.19 - 0.72	
Age	0.88**	0.98**	0.87**
95% confidence interval	(0.81 - 0.97)	(0.97 - 0.998)	(0.8 - 0.96)

Population average models with robust standard errors
* p<0.05, **p<0.01
Blank cells indicate variable not included in final model

7.3 Testing the hypotheses

Table 9 below shows how the analyses presented in this quantitative section triangulate in testing the hypotheses listed in section 4. In most cases, where a hypothesis was not supported, this was because the differences between categories of the variable of interest were not significant. However, for ethnicity, there was a significant association in the opposite direction to that suggested in the US literature for Exit1 in all methods of analysis, and for Exit3 in bivariate analysis and logistic regression. These analyses suggested that members of an ethnic minority were less, and not more likely to exit early.

Several variables that were found to be significantly associated with Exit1 and Exit3 in bivariate analysis were not in multivariate analysis which took account of the influence of other variables. This is important in focusing attention on those characteristics which are most important in making people vulnerable to early exit. For example, it is possible that the apparent association between being male and exiting early at Exit1 and Exit3 is due to the higher proportion of men than women who are referred to treatment through the criminal justice system. Treatment

services may do well therefore to focus attention and resources on how to retain more criminal justice referred clients, rather than enhancing services for men in general.

Table 9: Results of tests of hypotheses

Hypotheses		Bivariate analysis	Multivariate analysis Logistic regression	Multilevel modelling (HLM)
1. That people are more likely to exit between assessment and treatment entry (Exit1) if they are...	Male		ß	ß
	Primary stimulant user		ß	ß
	Member of an ethnic minority	ß	ß	ß
	Homeless (no fixed abode)			ß
	Subject to longer waiting for triage			ß
	Younger		ß	
	Referred by the criminal justice system			ß
2. That people are more likely to exit between treatment entry and 30 days (Exit2) if they are...	Male	ß	ß	ß
	Primary stimulant user	ß	ß	ß
	Member of an ethnic minority	ß	ß	ß
	Homeless (no fixed abode)	ß	ß	ß
	Subject to longer waiting for triage	ß		ß
	Younger		ß	
	Not entering substitute prescribing			
	Referred by the criminal justice system	ß	ß	ß
3. That people are more likely to exit between assessment and 30 days in treatment (Exit3) if they are...	Male		ß	ß
	Primary stimulant user		ß	ß
	Member of an ethnic minority	ß	ß	ß
	Homeless (no fixed abode)			
	Younger			
	Referred by the criminal justice system			ß
4. That different factors predict early exit at Exit1 and Exit2				

supported by the analysis, ß not supported

It is more difficult to interpret the differences in the results of hypothesis tests between the two different forms of multivariate analysis[10], although it should be noted that HLM is considered the more appropriate method for analysing data, such as treatment monitoring records, that is nested at different levels (e.g. treatment agencies and individuals). Taken together, these analyses provide partial, but not full support for the hypotheses that CJS referral, younger age and being homeless are predictive of exit before treatment entry.

Results for hypotheses on Exit2 and Exit3 are more consistent. The only consistently significant variable in predicting exit between treatment entry and 30 days in treatment is the type of treatment; people in substitution treatment were consistently less likely to drop out early. Age was very nearly significant in predicting Exit2 in logistic regression, and was significant in the HLM model. So it seems highly likely that there is an effect of younger age in increasing the likelihood that people will leave treatment early. For any early exit between assessment and 30

[10] It should be noted that the failure to find a significant association in one or other of these analyses cannot prove that the variable has no influence. Rather, they test whether the apparent association is likely to have arisen by chance. Variables which are not significant in these models may be influential, but not so influential as to show a significant association in one or other of these models

days in treatment (Exit3) the consistently predictive variables were younger age and homelessness.

Table 9 also suggests that the fourth hypothesis, that different characteristics are associated with early exit before and after entry, was consistently supported. The only characteristic to be associated with both stage of early exit was age (in HLM) and waiting longer for triage (in logistic regression). However, it should be noted that data was not available at the Exit1 stage on the treatment that the person intended to enter or had been offered. So it was not possible to test whether clients heading for substitute prescription services are less likely to drop out before entry, as they are in the first month of treatment.

It should be noted that one variable that was not included in the original hypotheses but was present in the dataset and in the analyses was also consistently predictive of any early exit in all three forms of analysis. People who reported being a current injector were less likely to exit early than those who did not. This mode of drug use seemed to be more influential than the actual type of drug consumed in influencing early exit.

These quantitative results suggest that homelessness, not being a current injector, being young, and being referred by the criminal justice service are particularly important characteristics that are associated with early exit from treatment. The high variation in rates of early exit between agencies, the low rate of early exit from prescribing treatment and the finding in HLM that people are more likely to drop out in the first few days of treatment at agencies with high median waiting times for triage suggest that individuals' characteristics may not be as influential as the type (and quality) of service that is provided. The qualitative data enabled us to look at these characteristics of individuals and services in more depth.

8. Qualitative findings

This part of the report is based on our findings from the qualitative interviews with 53 service users, individual interviews with 16 workers and the Warwick focus group.

8.1 Characteristics of service users exiting early

In this section of the report, we discuss the characteristics of service users who exit from treatment services, based mainly on service workers' reports.

Drug using communities

Some workers described the status and excitement involved in drug taking. Workers reported that being part of the community that drug taking provides, delivers a positive source of identity for its participants and is intrinsic to its allure:

> *There's also the excitement out there on the streets, you know being part of the drug culture, you know, one never knows what's going to come along next. (Provincialworker3)*

The fact that drug use provides 'work', a social life and status for a group of offending young (mostly) men raises challenges for treatment services. According to a harm reduction service manager this sense of identity and community of the 'in-group' of users can be transferred to the drug treatment service. In his view, his service has become popularly identified with the predominantly white, working-class, opiate-using young men in their mid-twenties to thirties, who are its main service user group, thereby making the service less accessible to, for example younger, problem cannabis users:

> *If I had someone, 20 years old, struggling with cannabis use,...and I was going to offer them an appointment I would get them to avoid coming in here on a [day of the week] for instance.. there'll be maybe 40, 45 prescribing service users coming through, the waiting room gets very busy, and it becomes quite clearly an opiate service for the day. (Provincialworker 2)*

The apparent domination and popular identification of treatment services with opiate using men clearly has a negative impact on engagement for those who do not identify themselves as part of this particular community of drug users. This may discourage women, users of other drugs, users who are either younger or older than the usual client group, and those from other ethnic groups from contacting the service in the first place. For one long term cannabis using woman there was a stigma in being seen to be using a service associated with heroin users:

> *I don't want anyone to see me and say, oh, look, because then they start making assumptions, 'is she a smack-head?', you know. (JANE)*

Age

We found evidence therefore that *younger*, non-opiate users may be put off by a service that they perceive is for heroin users in their late twenties and early thirties. The age of the drug user may also be linked to their stage in a drug 'career' however and the drug they use:

> *Generally by 18, if they are using [opiates] they've been through quite a lot already, and they're generally quite mature I think…. (Metropolitanworker 6)*

While our quantitative data suggests that older clients are more likely to stay in treatment than their younger counterparts, in the view of one worker, older service users are not necessarily more likely to engage in treatment, since they may be very *ingrained* in their behaviour, emphasising that drug use is a way of life.

Ethnicity

The provincial DAT area where we conducted interviews is less ethnically diverse than the metropolitan DAT areas, with smaller minority ethnic communities who, according to workers are underrepresented as service users.

> *[there is a massive] Asian community in [*Provincial town*] and we've got an Asian development worker here ..You know, I've got a tiny percentage of Asian service users, tiny. (Provincialworker 1)*

In the metropolitan DAT areas, workers were on the whole less concerned that service users were not representative of minority ethnic communities:

> *This Drug Treatment Service is a very ethnically diverse treatment centre. I mean we serve a very ethnically diverse population. (Metropolitanworker 4)*

In one of the metropolitan DAT areas where research was carried out, 33% of those interviewed were from minority ethnic communities, compared to 35% in the general population and in another, just under 33% of those interviewed were from minority ethnic communities compared to 28.8% in the general population[11].

Although we can be fairly confident that we interviewed BME service users more or less in proportion to the general population, we are unclear as to the extent to which problematic drug use is *overrepresented* in these communities. It is an open question as to whether drug treatment services are engaging Black and Minority Ethnic service users in proportion to their rate of problematic drug use, even in metropolitan areas. A report for the Greater London Authority (Greater London Alcohol and Drug Alliance, 2003) stated that problem drug users who are from black and minority ethnic groups have difficulty in accessing drug services relevant to their needs, that many people from BME communities are unaware of the services that are on offer, and that for people who do not speak English as a first language, it is difficult to access information about drug services. Research carried out by the National Audit Office (2004) also indicates that drug services need to target black and minority ethnic drug users and cannot assume that the ethnic breakdown of their clients reflects the needs of the local population. The fact that the two researchers were both of white British ethnic origin may have meant that service users from other ethnic groups were less comfortable identifying either lack of ethnic diversity in drug workers or racism more generally as influencing their reason for disengaging from treatment services. Certainly some of the women interviewed stated (to the female researchers) that they preferred women drug workers. We cite an extract from an interview on page 38 where Carl, like several informants, talked about appreciating the empathy of a worker who had had direct experience of problematic drug use himself. Carl, who was a British Asian, says that this worker is also 'cool' and black, suggesting that that there is also a need for drug workers to reflect the ethnic groups and backgrounds of the service users with whom they work.

Women

[11] According to 2001 Census data

Workers stated that women, particularly those referred by the criminal justice system, were *more* likely to be retained in treatment than their male counterparts, once engaged. This is supported by Millar et al's (2004) study in which males were 1.5 times more likely than their female counterparts to drop out early, although our quantitative data suggested that gender was not a significant predictor of early exit.

Workers stated though that parents of young children are less likely to present themselves voluntarily for drug treatment. The problem of finding childcare combines with a reluctance to attend treatment services because of concerns that social services will be alerted to their drug use. Two clients mentioned problems with childcare that made it difficult for them to attend appointments. The fact that the service that they were attending had no crèche facilities meant that it was necessary either to bring the children with them or find someone to take care of them.

> I thought they was lovely and I really did enjoy going but at the same time I was referred and given the appointment, sort of through Social Services for me to go, they didn't arrange no childcare for me (Veronica)

Lack of childcare therefore acts as a barrier for women to access drug service treatment. The need for treatment services to take parents' childcare needs into account has been a finding of successive studies on this topic (Gilbert, 1997; Hunter & Powis, 1996; Marsh, D'Anino, & Smith, 2000; Powis, 1995). Equally, the finding that women with children fear that drug treatment services will alert social services to their drug use has been highlighted by previous research studies (Hunter & Powis, 1996; Swift, Copeland, & Hall, 1996; Taylor, 2000) - a well founded fear for more than one informant in our study - indicating the need for low threshold, open access drug treatment services and advice sessions for drug using parents.

One drugs outreach worker stated that women drug users generally make themselves less *visible* than their male counterparts and although they may be homeless for example, they are less likely to be on the street. Different sorts of outreach strategies including the availability of drug treatment services in non-stigmatising generic health and social care settings are therefore required to enable women to access drug treatment services. and particular strategies are needed to engage particular groups of hard to reach and vulnerable drug using women. According to a worker in a town in the provincial DAT area, where there is a large sex working population, sex workers - often homeless, not in receipt of benefits and leading highly nocturnal lifestyles - are a particularly difficult group to engage and retain in treatment services:

> *Working girls, it's just a massive, massive problem to hang on to them…. Because they're up all night and they sleep all day. (Provincialworker 5)*

Workers indicated that the experience of domestic violence and male-domination of services may also mean that women feel intimidated by drug treatment services. Thus while there is a growing awareness that women generally have been under-engaged as service users, patterns of gender inequality and disadvantage may unwittingly be reproduced in treatment services. One worker described the following scenario for example, in a structured day programme which highlights a potential barrier to women's involvement in treatment services:

> *sometimes they walk in, we've had one recently walk in, took one look and saw an ex-abusive boyfriend on the programme, we didn't see her again either. (Metropolitanworker 3)*

Services cannot therefore assume that they are providing a gender neutral, woman-friendly environment and need to consider different strategies of engaging and retaining the full range of women drug users.

Chaotic drug users and crack

The category of the *chaotic* drug user features strongly in drug treatment workers' characterisation of service users who are most difficult to engage and retain in drug treatment services. The notion of the chaotic drug user operates both as a description and an explanation for the drug user – often using crack – who is too disorganised and/or disturbed to attend appointments. When these accounts are analysed however it would seem firstly, that the lives of 'chaotic' drug users are not so much disorganised as organised in such a way that does not coincide with the opening hours of services and secondly, that there may be good reasons why such users disengage from services, amounting to rational non-compliance rather than *chaos*:

> *If they're a stimulant user then it's like, 'well what am I going to do? I'm going to come and talk to you… How are you going to make a difference to me? (Metropolitanworker 4)*

The effects of crack were described in the following way by a prescribing worker in a service where it was reported that crack use alongside opiate use was 'epidemic':

> *Crack's tough yeah, just because of the nature of it. It's flibbertigibbet, nocturnal, hungry, how can you sit still in a room when you're starving… they need the basic needs really. I've always thought a crack service needs to have a lot of toast and tea you know. (Provincialworker 5)*

In our quantitative analysis, those who reported that their primary drug was a stimulant were more likely to drop out early (although this effect was not significant in multivariate analyses taking other characteristics into account). Those who reported injecting drugs at treatment entry and those who received a substitute prescription were more likely to stay in treatment (even taking other characteristics into account). This again suggests that most drug services are better suited to the needs of the traditional, heroin dependent injecting drug user than to those of the newer population of problematic crack users, for whom no substitute prescription is yet widely available. But there are still ways in which services can retain these people. It has been suggested that rapid intake by staff who are knowledgeable about crack and services such as relaxation techniques, cognitive behavioural therapy, complementary therapies, longer opening hours and the provision of the basics (such as transport to treatment and food, as suggested above) are valuable for crack users and can help retain them in treatment (Bird, 2006; Harocopos, Dennis, Turnbull, Parsons, & Hough, 2003; NTA, 2002).

As well as crack use, 'chaos' was also attributed by staff to the life-styles of sex-workers whose largely nocturnal working patterns mean that they find it hard to attend – particularly morning – appointments. Workers' accounts of the characteristics of *chaotic* drug users suggest a gap in services and a particular need for services that target both stimulant users and nocturnal drug users, like sex workers. The latter point is supported by a recent research briefing published by the NTA (Bloor, Crome, Moss, Freeman, Okolo, & Astari, 2006)) of a longitudinal retrospective study of sex workers attending a specialist, fast-track drug service. The study found that sex workers were more likely to attend the prescribing service where there were shorter waiting times, where evening clinics were provided and where transport was provided by outreach workers. A sex worker interviewed for our study emphasised that specialist services for sex workers need to include as broad a range of services as possible including 'mug shot' publications of violent customers and free access to condoms.

Other non-opiate users

Workers stated that another group of drug users who are likely to disengage early from services are benzodiazepine, solvent and problem cannabis users for whom there are no pharmaceutical treatment options:

> *a benzo habit is the worst bone of contention for me of all. I don't know why but it's just they're pushed aside, you know, you're better off coming in the door with a heroin...habit than you are a benzodiazepine habit. (Provincialworker 4)*

It was suggested that when they realised that only psycho-social help was on offer for, for example, problematic benzodiazepine use, service users were more likely to disengage and buy it illicitly. This is perhaps another example of drug users' rational non-compliance and illustrates the need for a more diverse set of drug treatment services including the needs of a broader range of problem drug users.

Dual diagnosis

Workers thought that the influence of dual diagnosis of mental illness and problematic drug use on disengagement was not predictable:

> *Two extremes, either very dependent, turn up bang on time, needy, they're desperate needy people. Or the other extreme is they just never ever manage to get here because they're too unwell. (Provincialworker 5)*

Neither was dual diagnosis an intrinsic barrier to treatment success. This rather relied upon workers who were adequately skilled. Dual diagnosis service users might however suffer from service overload and drop out for this reason:

> *I've got my appointment on medication, I have got the outreach coming out, I have got to go the Health Service Unit, why the hell do I have to come down and see you as well. (Metropolitanworker 4)*

A lack of joined up working between mental health and drug treatment services was described and a frustration that mental health services tend to refuse treatment to those they see as primarily having a drug problem.

No Shows

Workers reported that it was very common for their clients to not turn up to appointments, especially the first one:

> *[Somebody not turning up for their first appointment]'s very frequent as well. I would say that happens probably about 30% of cases. (Provincialworker 1)*

Other situations, such as domestic worries, poor accommodation or financial concerns also affected a service user's ability to attend appointments because they had too much else going on in their lives:

> ***The benefits system, the way it's set up at the moment, is a huge huge drawback, we quite frequently lose people in the first month because they still haven't been paid anything. (Metropolitanworker 3)***

However, as referred to below, workers did not immediately close the service user's case, but made varying degrees of attempts to contact service users by telephone or letter. Flexibility is an important factor, particularly in the initial stages of contact. One worker from an outreach service felt that their rate of service user engagement was good because of their ability to be flexible:

I think we do have quite good engagement rates because we go out to them, we are quite flexible about appointments being cancelled (Metropolitanworker 6)

8.2 Referral and beyond

Although it is not always easy to pinpoint exactly why some service users leave treatment between the referral and treatment entry, it is clear from our quantitative data and other studies that the highest rate of attrition is at this point. The referral process is therefore a particularly sensitive stage and one that needs to be handled carefully in order to encourage potential service users to engage. In this section, we discuss the referral process and factors that may affect engagement.

Reason for initial engagement to services

Just under one third of our fifty-three interviewees had been referred to treatment services via the criminal justice system (including court orders, probation, arrest referral or CARAT services in prison). Most of the rest had self-referred to treatment, having made contact with services either through their own research or on the recommendation of friends and acquaintances. Irrespective of the way in which service users were referred, most of the interviewees had decided to go ahead with the referral for their own personal reasons; even those who had to attend services because of a court order had come to the conclusion that treatment was a sensible option. For some, it was simply that they had reached a 'turning point' in their life and it was time to change:

Partly I wanted to try it, I…you wouldn't know it to look at me, but I had a good education, I've got a degree and I had a good job at one time until the drugs took over and I'd like to get back to that position. (CARL)

Others felt put under pressure to attend services because of family commitments or other influences:

I feel like they [social services] have got a gun to my head at the moment, maybe other users don't have that goal. (PATRICE)

Mum… kept pressuring me to go there and eventually I just thought 'yeah it's probably a good idea if I do'. (DENNIS)

A handful of those who were referred by the criminal justice system reported that they only contacted treatment because it was part of their order and that this lack of motivation led to their drop out from services:

I mean I went there for the wrong reasons anyway because I got sent in by a court. So my frame of mind was, I am being forced to come here. (TOMMY)

Most interviewees may have been willing to be referred to services in the first instance, but their expectations of the service were not necessarily met, and they thus dropped out at a later date.

Prior knowledge of services

Several interviewees had previously been in treatment, many more than once, and so had prior knowledge of what to expect of services in general. However, few knew exactly what to expect from the actual service that they went to:

> *They didn't tell me anything about it…Like all they said is they are going to drug test me…but they didn't tell me how it works. (FRED)*

This quote comes from a service user who was referred to services from the CJS. Bearing in mind that such service users are supposed to have given informed consent to enter treatment, it seems strange that referrers often did not communicate a clear idea of what the drug user can expect to get from a treatment service. Just one service user mentioned that he knew what to expect from the service he went to, because many of his friends had previously been there and *everyone who told me about it said that it was good; I never heard a bad word about it. (ANDREW)*. These accounts suggest that candidates for CJS referral are not adequately prepared for day programmes by either drug treatment services or the probation service.

It appears from the interviews that previous experiences in treatment and comments from other users can have a big impact on service users' expectations from services, which can colour their impressions quite considerably. A number of service users interviewed, particularly in the metropolitan DAT areas, who had dropped out of treatment three or more times in the past, had great difficulty in describing their most recent exit from treatment, because all the episodes overlapped in their memory and one bad experience led to all experiences seeming bad. Others seemed to have difficulty in separating truth from fiction; in other words, they seemed to use the conventional wisdom of other drug users about drugs services. Such word-of-mouth information about drug services is often exaggerated or out-of-date. Common themes of this nature included long waiting times, difficulty in accessing treatment and lack of support.

8.3 Service users' reasons for disengagement

In the light of some service users' multiple treatment episodes and the drug users' conventional wisdom about drugs services; it is often difficult for researchers to separate past from present and fact from fiction in drug users' accounts of why they have exited early from treatment. However, in the same way though that we raised the question as to whether the concept of *chaos* in drug-use could be re-cast in terms of rational non-compliance, we are interested in taking seriously drug users' own reasons for disengagement. What follows are the main reasons that service users gave for exiting services, juxtaposed where relevant to workers' views. For ease of reference, we have divided the reasons up into two sections related to the service user and to the service.

8.3.1 Service user related reasons for disengagement

Clients reported that they had disengaged from treatment services for a variety of reasons in their own lives. Sometimes former service users had simply moved away. Others has breached court orders and returned to custody. A small number had become abstinent whilst in prison and although they had made contact with treatment services when they came out, they did not feel the need for further support. For employed service users, full-time work often made it difficult to attend appointments; one service user pointed out that treatment services seemed to be designed exclusively for unemployed people. Other service users reported that they had disengaged because they were already in touch with a variety of other support services - including mental health or post tier-four after-care - and did not also need the support of that treatment agency.

Coping alone

A small number of former service users interviewed had decided to deal with their drug problems on their own, without the help of services. One former heroin user for example reported that he had briefly attended a drug treatment service and, because he found the experience uncomfortable and stressful had '*decided to cluck it out on my own*' (JOE)

Four heroin users described having attended private clinics for prescriptions, detoxification and residential rehabilitation. One service user who was unable to get buprenorphine through the NHS was buying it through the internet. Based on these data, it is important to note that drug users do use alternatives to NHS and voluntary sector drug treatment services. They can resort to the market (both legal and illicit) in private treatment services. While clearly attending private clinics is not an economic option for all drug users, it does challenge the notion that exit from drug treatment services necessarily indicates a failure of 'motivation' on the part of users to address their drug problems.

Motivation

In order to explain their disengagement from treatment services, service users drew on a number of drug treatment discourses, including, for example, that responsibility for drug misuse and disengagement lies with the individual alone. The use of professional discourse on motivation to explain exit (by both workers and clients) can have a self-fulfilling effect. Explanations for disengagement that rely on the lack of client motivation place the onus on the individual service user, rather than seeing the process of engagement and disengagement as a dynamic interaction between service user and the treatment agency. A large number of former clients firmly located the reason for their disengagement with themselves rather than any fault of the treatment agency: *'Yes I can't fault the service.. it's just down to me and where I am, the decisions I make' (CRAIG)*

While service users often ascribe early exit to their own lack of motivation, it would seem from our data (and from the research literature) that motivation can be developed or discouraged by the actions of treatment services. The latter point is reflected in service users' descriptions of the sequences of events that led them to drop out. This included the examples of appointments being cancelled by drug services and of the hurdles that drug users perceived as blocking their path into treatment. A long-term heroin user in his fifties described the disappointment of getting himself to the point where he was ready to accept treatment only to be confronted with multiple assessments and waiting lists for treatment:

> *… it's hard enough to like after 30 years to think about stopping, to be told you've got to come back for this and that and that (DESMOND)*

Motivation to change behaviour and engage with treatment services is therefore fragile and easily dented. First impressions of the treatment experience, multiple assessments, the skill of practitioners and the length of waiting lists are thus crucial components in retention and engagement.

Our interviews indicate that service users' motivation to engage in and be retained in treatment services can be both encouraged and discouraged by attitudes and practices of treatment service staff. While it may be the case that responsibility for disengagement often lies with the service user, our data reinforce research that shows that motivation can be nurtured by drug services and is not just a characteristic that service users bring to treatment (Miller, Rollnick, & Conforti, 2002). Indeed, as Fiorentine et al (1999) have suggested service user receptive services are more important predictors of positive outcomes than treatment receptive clients.

Chasing Service Users

One way in which drug treatment services can develop motivation is by 'chasing' service users who do not attend. Although proactive, personalised contacting of service users has been found to increase retention (Ashton, 2004) our focus group participants thought it was not

feasible to send hand-written letters to service users who did not attend initial assessment interviews in large and busy services.

How proactive services are in attempting to make contact with non-attenders appears to vary from a 'standard letter' to repeated calls to mobile phones:

> *I got a letter saying that because I failed to go and meet their requirements I was off their books but if I intended to try again in the future to please get in touch and they would offer me all the help that they could.* (MARTIN)

Prescribing services were more persistent in their attempts to get service users to appointments. The following extract describes the successful effects of such a strategy.

> *A couple of them [letters and phone calls] I ignored because at the time I was on drugs and whatever but then after a little while I did actually contact them because I wanted to get back in treatment and they helped me the same as they did before.* (AMY)

8.3.2 Service related reasons for disengagement

A number of factors relating to early exit are under the control of drug treatment agencies. These included issues such as bureaucracy, waiting times and the lack of treatment options. There was some overlap here with the views of workers; however, in other cases, the views of workers and service users were completely different. For example, service users were specifically asked if the setting of the service itself (specifically the state of repair and layout of the building) had a negative impact on them; very few believed that this was a problem. However, several workers brought this up as an issue.

Workers were often aware of factors that deterred service users from remaining in treatment, yet felt that because of resource implications, organisational structures or bureaucracy imposed by senior management, solutions were outside their control.

First impressions of service

The vast majority of service users were relatively positive about their first visit and mentioned that both the staff and the building gave a good first impression; in fact, just three service users had a negative reaction and this was related as much to the stigma of the building as a drug treatment service, as it was to its appearance.

One service user reported that she felt that the state of the building, the most neglected of the metropolitan services visited by researchers, made her feel more at home:

> *…in a way, it's good that it's not all new and everything because it just seems easier, more like us really, run down and that…(MAGGIE)*

For our focus group participants, the quality and layout of the building was felt to impact on service user engagement. This group of staff also felt that security arrangements that require those seeking to enter to press a buzzer and announce themselves through an intercom and where reception staff are often behind screens were a barrier to engagement.

It is an open question however whether those service users for whom scruffy premises and/or security are a barrier to engagement ever get over the threshold of the buildings, and indeed whether the quality of the building is more demoralising for treatment service staff than for service users.

However, of those who had largely positive first impressions, not all stayed that way, feeling that once they had been assessed, the good service came to an end. Although research shows that a continuing good relationship with staff – the therapeutic alliance - is important for retention; the following quote illustrates that support from staff is not always maintained, at least not to the satisfaction of the service user:

> *When you first go in they seem really supportive and all that. You think 'yeah, I've got a bit of hope here'. But as soon as they book you in, that's it. You're just left alone. (TOMMY, 37)*

Service users' knowledge can also play a major role in their first impressions of a service. Although many service users had previous experience of treatment, in the view of staff, many are still not fully aware of what they can and cannot expect, which can lead to disappointment. Referring agencies therefore need to be made clear as to the sort of service that is available from drug treatment services. Focus group staff participants also reinforced the point that service users need a clear introduction during the initial visit as to what they can expect.

Relationships with service staff

Many of the interviewees had negative comments to make about service staff, particularly their key worker. This was often because the service user felt that their key worker had no experience of using drugs and thus did not understand them:

> *…sometimes you feel like belittled if you like. You know and you usually find that's from people who haven't used themselves. (DONALD)*

Others simply felt that the worker was not responsive to their needs:

> *You know, you always get the same excuse, 'oh I'm a bit tied down in paperwork', you know, or 'come and see me in an hour' or 'get the doctor'. They think the problem's going to go away. (TOMMY)*

Two service users felt that workers were overloaded because of lack of staff. However, understaffed or not, the amount of time that a key worker spent with his/her service user and the extent to which they listened to their needs in an empathetic way was noticed by service users and was reported to have had an impact on their willingness to stay in treatment. Good relations with a key worker often encouraged service users to stay in services; in some cases, service users who had built up a rapport with staff before dropping out were willing to return to the service if the same worker was available:

> *I really liked my keyworker ... He was dead cool. He was black and an ex-user, he had all sorts of suggestions of ways of staying off the gear, I just really liked and respected him. I've never met anyone like him since – I've been really lucky he's still there and I've managed to get back with him. (CARL)*

In the view of focus group participants, lower service user to staff ratios make a difference to the quality of treatment service offered. They reported that it improves service user retention and enables more outreach activities with harder to reach drug users.

In their recent qualitative study of user involvement in Scotland and England, Fischer et al emphasise the importance to service users of key workers who are empathetic and responsive to individual service users' needs (Fischer, Jenkins, Bloor et al., 2007). Our data suggest, in line with previous research (e.g. Fiorentine, Nakashima, & Anglin, 1999; Petry, 1999), that good relations between key workers and service users are positively related to engagement and

retention, and may mean that even when service users disengage they are more likely to re-present for treatment.

Bureaucracy

Some staff raised the issue of bureaucracy and the long waits for treatment once the triage assessment has been carried out, which had a direct impact on the quality of service once the initial contact had been made.

> *… people's frustration is the unwieldy machine that seems to take forever. Now I know there's reasons for that, but I do question what the reasons are sometimes. (Provincialworker 6)*

The length and complexity of the assessment process was mentioned as an issue, although not necessarily one that would put service users off on its own, as well as the fact that the service users were often asked the same questions several times:

> *Now why that form can't be combined is beyond me, why can't it be pages 1 to 32, starts with triage, ends with comprehensive? Why have we got two forms? (Provincialworker 2)*

This was an issue that service users also brought up:

> *The assessment interview? I thought it was just totally stupid…they already knew all the stuff here that I was going to tell them (ALISDAIR)*

Waiting times

As discussed in the literature review, waiting times have implications for service users' exiting services, particularly before beginning treatment (Strang, Best, Ridge et al., 2004), although these are by no means the most important factor in early retention. Of the service users we interviewed, although waiting times were rarely the sole reason for leaving, it was often important. Waiting times clearly varied greatly across services; sometimes it was just a matter of days, other times, it was weeks:

> *…I come down, come for the appointment, got assessed, they said all right, we'll let you know in a few weeks time, you'll be OK in a few weeks time…so I would be either dead or in prison or out there, skinny as a rake, keeping my habit going. (BRIAN)*

Some of the waiting times described by service users clearly do not fit in with the NTA's targets which, for specialist prescribing is 3 weeks; for GP prescribing, 2 weeks; structured day care programme, 3 weeks and residential rehabilitation, 3 weeks. Some service users were aware that those coming through the CJS were able to access treatment far more quickly; one service user seeking a place in residential rehab had thought about committing a crime so that he could get a place:

> *I want to get in there as soon as possible, you know, and if I have to get myself nicked to do it, I will, but I don't really want to have to go down that avenue, do you know what I mean. (NEIL)*

The variation in waiting times between services can be quite extreme. Some services claim that they usually manage to get service users into treatment within the waiting time target; others claimed that because of resource constraints, there were simply too many service users and not enough staff to see them all during working hours:

> *[Waiting time for treatment] really depends on waiting lists, if they're going on perhaps a methadone script, or something like that, it can be four weeks, but it can be as quick as…four days, but it depends which part of [area name] you live, [place name] is a bit quicker than [place name] (Metropolitanworker 6)*

Most workers felt that waiting times, primarily those for treatment, were an issue that directly influenced service user satisfaction with services. This was usually a factor that was not under workers' control and they expressed their frustration with their inability to move any faster. In addition, some service users (especially in the metropolitan DAT areas) are not registered with a GP when they first attend services and services are unable to prescribe until service users are covered by primary care[12].

It seems likely that the unevenness of waiting times between different areas, services and groups of people will have an influence on drug users' willingness to contact and engage with treatment. Conventional wisdom in drug using social networks about long waiting lists may provide drug users with another reason to avoid treatment, especially if it is confirmed when they do contact treatment services.

Non-provision of service requested

Two of the most common complaints from service users who had not received the service that they wanted was the lack of funding for places in residential rehabilitation and the non-provision of the medication that the service user felt was most appropriate for his/her needs. Service users were generally aware that residential rehabilitation is expensive but still found it very off-putting to have to wait for so long:

> *… the referral [to residential rehab] was never made and I never got in there, and for about six months I was really on the edge, tried to commit suicide…(FRANK)*

Complaints were also made about the non-provision of buprenorphine. As described above, one former service user had failed to persuade his GP to prescribe him buprenorphine and purchased it himself over the Internet, disengaging from a service that he perceived could not help him.

Others from the same service reportedly asked for, but were refused, buprenorphine (also known as Subutex – the name under which buprenorphine is marketed):

> *I did specifically ask for Subutex when I came out of prison… but I couldn't get it (ANDREW)*

Comment on the relative effect of buprenorphine and methadone in blocking the effects of heroin, is commonly heard from heroin users. They tend to report that buprenorphine blocks the effect of heroin, whereas methadone does not. This is despite the agonist effect of methadone, which means that it blocks the effects of heroin if an adequate dose is taken. The effective maintenance dose of methadone is considered to be above 60 mg per day, but 75% of primary care prescriptions are for less than 40 mg (Strang, Sheridan, Hunt, Kerr, Gerada, & Pringle, 2005). In order to reduce risk of causing death through overdose, treatment usually starts with low doses which may be increased if the patient displays tolerance. The majority of people who start a methadone prescription therefore do not initially receive a dose that is sufficient to block the effects of heroin, and it may take some time to increase the dose to the level they feel comfortable with.

---

[12] This appears to be a local policy decision of prescribing services involved in this study rather than a national policy position

> *When they originally started me on the methadone, they started me on 30mls and it was not enough, so I was using on top of the methadone…their policy is 5mls [increase] a week…it wasn't doing anything. (MARTIN)*

In contrast, typical initial doses of buprenorphine (which is safer in overdose than methadone) are, according to users' reports, effective in blocking heroin. So it seems that clinical decisions on methadone titration, rather than just the pharmacology of the drug, contribute to the preference for buprenorphine among many heroin users. Alternative clinical practices, such as relying on drug users' reports of the methadone dose that they need (with some safeguards) may enhance retention (Bakker & Fazey, 2006) although, as noted in the literature review above, this practice is regarded as dangerous by others in the field who argue that clinical attention should be focused on the optimum maintenance dose of methadone rather than its induction dose (Zador & Farrell, 2006).

According to workers, the relative expense of buprenorphine (compared to the cheaper methadone) means that there are limits on the amount that can be provided and increasingly strict criteria for its prescription

> *if you're using more than a couple of bags a day you're unlikely to get Subutex (Provincialworker 4)*

However, a worker from a service, reportedly suffering from severe resource cut-backs, claimed that buprenorphine was their preferred drug. It is thus easy to see how service users become frustrated with service provision when there is apparently so little consistency in treatment options.

In the same way that opiate users report exiting treatment where they have found bruprenorphine is not available, as we described in relation to workers' accounts, stimulant users, solvent users, problem cannabis users, users of benzodiazepines and other problem users of prescription drugs for whom there is no pharmacological alternative also reported exiting early because of a lack of convincing or *motivating* treatment options. This was expressed in the following way by one long term benzodiazepine user:

> *I just couldn't get off my arse to go to tell you the truth* (ENID)

This quotation also illustrates the sorts of barriers to engagement that service users generate and what drug workers are up against in their attempts to engage clients; recognition that their drug use has become problematic does not necessarily translate into *motivation* and engagement with services.

Service users also described the supervised consumption of opiate substitutes as uncomfortable and stigmatising:

> *I didn't like the attitude of the people in the chemists, they didn't really want me there and I felt uncomfortable. Sometimes I felt so bad I came out and went to use on top* (NIGEL)

In addition, service users raised the problem of the restrictions on numbers of substitute prescription customers that pharmacies can accept at any one time. This is a problem in more rural parts of the provincial DAT area, where, when the local pharmacy is 'full', service users may have to travel some distance in order to access their medication.

For one homeless drug and alcohol user who had been a service user of an outreach drug service, his drug use was simply not his first priority, but the service he attended was unable to help him:

> *He was more bothered about me getting treatment for my drug use. I couldn't understand why - I kept telling him 'get me housed and then I'll think about the drugs'. (NIGEL)*

Services need to build links with housing and other organisations in order to help service users who have problems that are not directly associated with their drug use.

Mixing non-users and users

The fact that most services in our sample did not differentiate between those who had stopped using drugs and those who were continuing to use was a problem for some interviewees, who found that mixing with service users who were still using was distracting and encouraged relapse.

> *I'm trying to come off drugs, I don't want to be in a room with a load of other drug addicts…I just didn't really want to see or know any other drug users. But it didn't work because it was quite a small place. (ANTHONY)*

Some interviewees also mentioned other service users using on site:

> *… they sit there five minutes smoking their crack then they come out and go and have their appointment, their interviews and then they get their scripts and then they are off for another week. (MARTIN)*

Four service users, all younger, non-opiate users from the provincial DAT area, mentioned that they felt uncomfortable in a service dominated by offending, opiate users whose drug use they saw as far more serious than their own.

Dislike of groups

Other former service users had more negative reasons for disengaging that related to their personal response to the sorts of service available. A common theme was a dislike of groups and counselling more generally. Sometimes this was because former service users felt uncomfortable talking about their drug problems in this forum.

Occasionally service users had been made uncomfortable or even threatened in a group with somebody they knew to have benefited materially from others' drug problems:

> *These are the guys I used to give my money to and they're in a group there, you know talking about their problems…that was winding me up a little bit. (ROGER)*

Other former users felt uncomfortable talking about their drug problems even in one-to-one counselling sessions. For some, this was expressed as a concern about privacy and information-sharing - *'I don't like talking about my drug problems with other people, don't like other people knowing about my drug problems'* (JOE)

Unsurprisingly perhaps, the social relations of the illicit drugs economy may also be reproduced within a day programme where someone may find themselves sitting next to their dealer in the supposedly 'safe' environment of a group therapy session. Our findings emphasise that in order to maximise engagement and retention and reduce stigma, treatment services need to both be provided in more generic health and social care settings and in such a way that targets particular groups of hard to reach and vulnerable drug users. Our analysis highlights the fact that drug users, including those involved in criminal activities, sex work and addicted to crack cocaine have organised lives and are rational actors. We therefore question the notion of the chaotic drug user and argue that services need to be adapted to the needs of hard to

reach users. Our findings about the importance for service users of empathetic staff, their desire to exercise choice about the sorts of services they receive and the necessity for treatment services to be flexible enough to meet individual client needs support the findings of the recent study of user involvement by Fischer et al (Fischer, Jenkins, Bloor et al., 2007)

Finding the solution to some of the problems with the organisation and provision of drug treatment services in order to minimise early exit will in some cases have resource implications. In some, however, including the provision of a wider range of low threshold, drop-in and counselling services in generic health and social care services such as GP surgeries may not require new resources. As Godfrey, Stewart and Gossop (2004) have shown the estimated benefit:cost ratio of successful retention in treatment services is very high.

9. Discussion

This study included a mixture of quantitative and qualitative methods. In this section we discuss how the findings of these methods converge or diverge, as well as their limitations in answering the questions we have sought to address. It also discusses the needs for further research that we have identified.

9.1 Comparing methods

In general, our quantitative findings on the kinds of drug users who were most likely to drop out early (i.e. those who were young, homeless, criminal justice referred or not injectors) link well with our qualitative data, which suggested that the sampled services tend to be focused on the needs of the people who could be described as the traditional client group of drug treatment services; injecting opiate users in their late twenties and thirties. Other drug users, who have different needs and are involved in different social networks may perceive such services as intimidating and excluding. There is some rationale to this, given the history of the English drug problem, which has long been associated with heroin use and, more recently, with the threats of HIV and Hepatitis C to injecting drug users. It is also true that drug treatment services may have, in the form of opiate substitution drugs, more to offer heroin injectors who wish to move to less dangerous forms of drug use. Nevertheless, as 59% of problematic drug users have recently been estimated to be users of crack (Hay, Gannon, MacDougall, Millar, Eastwood, McKeganey et al., 2006) and attention focuses on the needs of younger drug users, who are more likely to have problems with cannabis, it is increasingly important that drug treatment agencies develop services that can attract and retain people outside the traditional client group.

For some homeless and no fixed abode drug users, consistent attendance at drug service appointments may raise practical difficulties and represent less of a priority compared to overwhelming housing needs. Particularly in the provincial DAT region, the perception that services are primarily oriented to opiate using men in their late twenties and early thirties seemed to deter younger and non-opiate users, both male and female from engaging with services.

There were other themes on which the quantitative and qualitative analyses seemed to concur. One was the apparently greater likelihood of early exit among men, although, as discussed above, this may be due to the greater proportion of CJS referred clients among men than women. Our quantitative analysis suggested that people who were referred by the criminal justice system were more likely to drop out between assessment and treatment entry, but not between entry and 30 days in treatment. Our qualitative interviews with people who had been referred in this way supported the findings of other research ((Stevens, Berto, Frick et al., 2006) that being involved with the CJS does not necessarily mean that drug users have lower readiness to change than other treatment clients. The high rate of drop-out after assessment suggests that there may be an issue of some criminal justice involved drug users being referred for inappropriate referrals, when they have expressed no willingness or need to enter treatment. This was found, for example, in the recently published evaluation of the Restriction on Bail pilot (Hucklesby, Eastwood, Seddon, & Spriggs, 2007). It may also be the case that CJS involved clients need more intensive support to get them from assessment to treatment entry, as they are likely to be dealing with several issues at once, including legal, medical and housing needs, as well as treatment needs. Services that have been made available to offenders, such as rapid entry to treatment, may therefore enhance their early retention.

Our two data sources also both suggest that some agencies are better than others at getting the basics right; i.e. providing a positive, welcoming environment to which drug service users wish to return once they have first encountered it. Once these basics are addressed, systematic use of motivational interviewing, which was not widespread in our sampled areas, and of other counselling enhancements, such as node link mapping[13] and contingency management[14], offer the prospect of further improvements in retention rates.

Perhaps the most important area of concurrence between quantitative and qualitative methods was on the sheer scale of the problem. The quantitative analysis suggested that nearly a quarter of people who contact drug treatment services and are assessed in our sampled areas do not go on to last a month in treatment, with over two-thirds of this drop out occurring between assessment and treatment entry. This fitted with reports in staff interviews of the high frequency of clients not turning up, especially for the first appointment. It also fitted with our drug user interviewees reports of multiple, and often short periods in contact with treatment. If drug treatment services are to maximise the opportunities afforded by the impressive increase in the numbers of people who are in contact with drug treatment, then they will have to find ways in which to ensure that this contact lasts long enough to have an effect on the health, offending and other problems of dependent drug users.

One possible solution to the perceived domination of an in-group of drug users and associated features (such as the presence at drug treatment centres of people who are known as former dealers or victimisers) which make a service less accessible for others, is for there to be diversification of service location, including outreach/peripatetic working and a broader range of drugs services available in GP surgeries and health centres. This would enable drug users to contact professionals who could help them without having to go to a location that is perceived as excluding and with less risk of coming across people whom they fear or who may act as triggers to relapse. Another possible solution to the needs of drug users who are perceived to be "chaotic" is to provide open access to services that are open during the hours that they are likely to be needed (i.e. at night, as well as daytime). Both potential solutions pose challenges to the traditional pattern of providing drug services from centralised locations during office hours. Workers and managers with whom we discussed these potential solutions recognised the need for them. But they warned of how difficult it may be to make such changes, which are likely to require to changes in both commissioning frameworks and staff working practices.

9.2 Limitations

As in every study, there are a number of limitations that should be borne in mind when reading and using these results. They include the generalisability of the findings from relatively small samples to the wider population of drug users and treatment agencies, the potential for recording practices to affect the quantitative data and our failure to recruit a large group of members of ethnic minority drug users for qualitative analysis.

Although our sample of over 2,500 people entering drug treatment services is large enough to enable powerful statistical analyses of the influences on early exit[15], it is small compared to the

---

13 Node link mapping is a technique which involves the counsellor and client creating a diagram of thoughts, feelings and actions and how they are linked. It has been found to increase client engagement and retention in treatment (Simpson, Joe, Rowan-Szal, & Greener, 1997)

14 Contingency management involves the use of prizes, vouchers and/or clinic privileges in order to reward and incentivise good progress in treatment. It has been found, in several US studies, to improve retention and outcome (NICE, 2007)

15 For example, we calculated the statistical power of our logistic regression analysis on the basis of a formula provided by Hsieh et al (1998). Assuming that 25% of the sample exits early and that the minimum size of odds ratio that we are interested in is 1.3, with a confidence level of 95%, then the minimum sample size to achieve statistical power of 80% would be 1,432. This was comfortably exceeded by the size of the sample we used.

181,000 people who entered drug treatment services in 2005/6. It is important to note that the DAT areas that were sampled had lower than average 12 week retention rates. This means that the reported rate of early exit from these areas is likely to be higher than that for all DAT areas. Nevertheless, the rates reported here indicate the large potential for improving longer term retention and outcome by improving retention in the first few days and weeks of treatment.

The research design incorporated both provincial and metropolitan areas in order not to distort the analyses by excluding one or other type of area, but the sampled areas may also have features and patterns of drug treatment services that are not shared by other areas. This problem is more acute for the qualitative sample, which included only 53 service users from 10 agencies. Although we considered that we achieved data saturation on most theoretical stratifications of interest (except ethnicity) within this sample, the practices and experiences of clients and workers at these agencies may be different to those at other agencies.

We relied on the NDTMS data in our quantitative analysis and were not able, as we had hoped, to triangulate this data with data held in agency casefiles. The NTA has put much effort into improving the validity of the NDTMS data in recent years. However, it is still possible that recording practices vary between agencies and between workers in the same agency. The use of HLM should control for any systematic differences between agencies, but it should still be recognised that the data we used is produced through a social process with its own pattern of distortions and is not an exact facsimile of reality. For these reasons, our findings should be considered as suggestive and not a definitive description of the pattern of early exit from English drug treatment agencies.

A limitation of which we are particularly conscious is the lack of qualitative information on the influence of ethnic origin on early exit. We designed specific methods to recruit members of ethnic minority groups to our qualitative sample and followed them through. Even with these specific methods, we recruited only 13 members of ethnic minorities. Although this is an over-representation relative to the proportion of ethnic minority members of the quantitative sample, it was still too small to achieve data saturation. The fact that the research team were all of white British ethnic origin may have played a part in our difficulties in recruiting participants of other ethnicities. Interviews with the people we did recruit from ethnic minorities did not suggest that there were particular issues to do with ethnic minority origin in explaining early exit (as also suggested in the quantitative analysis), but this finding is open to challenge by other research that is able to focus more specifically on these groups.

9.3 Further research

All feasible studies are limited in their scope, and all discover valuable questions that they are not able to answer. We have uncovered aspects of drug service users, and of treatment agencies that deserve further attention in attempts to reduce early exit from drug treatment. Such further examination should involve:

- Comparative research on the practices (including recording practices), staffing levels and caseloads of those agencies with high reported retention rates, compared to those with low retention rates.
- Longitudinal research that is able to follow drug users through several episodes of treatment, in order to understand the cumulative effects of various stages in the treatment journey.
- Ethnographic research among groups of drug users to understand the flow of information between drug users about drug services and the effects of diversity, stigma and fear (of other drug users and of losing children to local authority care) within drug using sub-cultures on the decision to enter and stay in treatment.

- Experimental, or quasi-experimental studies that test the effects in practice (including the cost-effectiveness) of the improvements that we suggest in the conclusion.

10. Conclusions

From our analysis of the date we gathered, we offer the following conclusions on estimating, explaining and reducing early exit.

10.1 Estimating early exit

In the sampled drug treatment areas, in 2005/6, the proportion of people in contact with structured drug treatment clients who exited between assessment and 30 days in treatment was 24.5%. More than two thirds of this early exit occurred between assessment and entry into treatment.

However, there were wide disparities in rates of early exit between treatment agencies. We calculated, using logistic regression to control the influence of the service user characteristics, that the predicted probability of a person exiting between assessment and 30 days in treatment was 4.9% at the best performing agencies compared to 65.8% at the worst.

From our qualitative data, it is likely that some of this early exit represent the attempts of problematic drug users to 'go it alone' rather than engage in treatment, but it still seems that high rates of early exit represent a waste of resources and opportunities to change lives for the better.

10.2 Explaining early exit

The characteristics of service users that were consistently associated with being more likely to exit *between assessment and 30 days in treatment* were:

- Being younger
- Being homeless (of no fixed abode).

The following characteristics were associated with *exit between assessment and treatment entry* were:

- In logistic regression, being homeless and being referred by the criminal justice system.
- In multilevel modelling, being younger.

The characteristics that were associated with *exit between treatment entry and 30 days in treatment* were:

- In both logistic regression and multilevel modelling, being in a form of treatment other than substitute prescribing.
- In multilevel modelling, being younger.

Several treatment staff that we interviewed focused on characteristics of service users, such as the "chaos" of their lives and their lack of motivation in explaining why they leave treatment early. However, the existing research, our data on the different levels of early exit between agencies and the reports of other staff and service users whom we interviewed suggest that there is much that services can do to enhance the rate or retention in the first few days and weeks.

Drug users who do not belong to the traditional client group of injecting heroin users in their late twenties and thirties may find traditional drug services, provided from central locations in often run-down buildings, off-putting. The opening hours of services may exclude many potential clients, especially those who work (including those who work in the sex industry), from being able to attend treatment.

Some staff reported that they do make use of recommended techniques for enhancing early retention, such as proactive, personalised contacting for appointments and motivational interviewing during treatment sessions. However, the use of such techniques was not widely reported by staff or service user interviewees.

Our interviews with drug users also suggested that, by failing to publicise their services and to clarify expectations in advance of contacting treatment, treatment agencies leave the main source of information that drug users have about treatment to be the user's own previous (often unsuccessful) episodes of treatment or the conventional wisdom on drug treatment that is circulated through networks of drug users. It seems that clients referred by the criminal justice system may be particularly under-informed of what to expect from drug treatment.

There are things that drug services do that seem to deter some drug users from engaging in treatment. These include requiring drug users to go through repeated, lengthy assessment processes and multiple appointments to actually get treatment, not providing the treatment (especially residential rehabilitation and buprenorphine prescriptions) that some of our interviewees had hoped to get, insisting on supervised consumption of methadone, starting methadone prescription at doses that may be too low to help the drug user and mixing drug users who are at different stages of their 'treatment journey' in the same groupwork sessions.

10.3 Reducing early exit.

Our estimate of the scale of early exit suggests that this stage of the treatment journey offers scope for much improvement in the quality and effectiveness of drug treatment. We offer the following recommendations that may contribute to such an improvement. They should be seen as suggestions that can be tested through examination of current services that already follow them and future innovations in service delivery.

1. As the current pattern of service delivery can be excluding to many problematic drug users, we recommend that there is greater diversification of services in terms of locations and opening hours. This could involve the expansion of shared care in primary care locations such as GP surgeries and other community settings to include psycho-social care and brief interventions as well as maintenance prescriptions. In addition there should be an expansion of peripatetic and assertive outreach services for women with children and other harder to reach drug users (including younger drug users and sex workers).

2. As many drug users seem to be using inaccurate or out-of-date information about treatment when they decide whether to engage in it, we recommend that treatment agencies make a greater effort to describe and publicise their services to potential users. This requires a change in the culture of services that are used more to responding to demand than to creating it.

3. As problematic drug users have reported that inflexible frameworks for prescription contribute to early exit, we recommend that services offer more flexibility in prescribing (e.g. wider availability of buprenorphine and rapid prescribing)

4. There is a need for further examination of the safety and retention rate associated with methadone tolerance testing. If safety concerns can be dealt with, this offers a potentially valuable method for overcoming many of the complaints (e.g. waits for treatment, repeated assessments before treatment) that were expressed by our opiate-dependent interviewees.

5. As some of our interviewees reported that the stigmatisation and inconvenience involved in supervised consumption of substitute drugs influenced their decision to

leave early, consideration should be given to reducing these deterrents by offering privacy and flexibility in location and time of consumption. People on supervised consumption should always be able to consume their medicine without being identified by other pharmacy customers as drug users.

6. Childcare remains an issue that prevents many problematic drug using parents from engaging in treatment. So we recommend that services take on existing recommendations to actively encourage parents with young children to engage in treatment.

7. We recommend that waiting times are reduced at those agencies which are still missing the targets by increasing staff:client ratios and by ensuring that sufficient pharmacy slots are available.

8. Given the proven effectiveness of motivational interviewing in enhancing motivation and retention, we recommend that there is more use of motivational interviewing early in treatment. This is particularly important in view of programmes to divert offenders from the criminal justice system into services, where motivation may be particularly tenuous and mutable.

9. We recommend that services adapt assessment processes so that they are consistent with the development rather than destruction of such tenuous motivation. Processes in general should be as simple and clear as possible so that drug users do not have to negotiate several stages of bureaucracy in order to access treatment.

10. We recommend that services take on existing recommendations about proactive, personalised outreach during the waiting time and in response to non-attendance. This would help to demonstrate the welcoming attitude which should be maintained throughout the service users' episode in treatment.

11. Specifically for crack users, and subject to the forthcoming findings of NTA sponsored research on crack services, we suggest that there should be rapid intake into treatment by staff who are knowledgeable about crack and that services such as relaxation techniques, cognitive behavioural therapy, complementary therapies, longer opening hours and the provision of food and transport may assist early retention.

12. Homeless drug users are another important group. Our quantitative data suggests that they are particularly vulnerable to early exit. Services that can rapidly assist with housing are more likely to retain such people. This points to the need for drug treatment services to deal with the wider issues, including welfare benefits and GP registration, that can block people from entering and staying in treatment.

We recognise that many of these recommendations imply increased expenditure on drug treatment services. However, the estimated benefit:cost ratio of such services is very high (Godfrey, Stewart, & Gossop, 2004). Increases in cost that lead to increased retention and effectiveness are likely to prove cost-effective, even if they do increase the unit cost of treatment. This, of course, is another suggestion that awaits empirical verification.

This report has estimated the rate of early exit from treatment, has identified some characteristics of drug users and services that are useful in explaining early exit and has made recommendations for how services may be able to reduce the rate of early exit in order to increase the quality and effectiveness of drug treatment. It is open to challenge or support by further research on the same issues. We hope that it will prove useful to policy makers and practitioners in the field.

Acknowledgements

We would like to thank the participating drug services and interviewees for their contribution to this research, as well as Beryl Poole for her specialist advice and Isobel Kessler for her contribution to the early stages of the project.

All errors and opinions are the responsibility of the authors.

Potential conflict of interest: Neil Hunt is partly employed by one of the agencies that provided interviewees. However, his input was mainly at the research design stage. He did not play a direct role in analysis of data.

References

Ashton, M. (2004). The power of the welcoming reminder. *Drug and Alcohol Findings*, 11, 4-19.

Atkinson, R., & Flint, J. (2001). *Accessing hidden and hard-to-reach populations: snowball research strategies. Social Research Update Issue 33* Guildford: University of Surrey

Bakker, A., & Fazey, C. (2006). Methadone Tolerance Testing in Drug Misusers. *BMJ*, 333, 1056-1059.

Beynon, C., Bellis, M., & McVeigh, J. (2006a). Trends in drop out, drug free discharge and rates of re-presentation: a retrospective cohort study of drug treatment clients in the North West of England. *BMC Public Health*, 6(1), 205.

Beynon, C., Bellis, M., & McVeigh, J. (2006b). *Trends in drop out, drug free discharge and rates of re-presentation: a retrospective cohort study of drug treatment clients in the North West of England* Liverpool: Liverpool John Moores University

Bird, M. (2006). Presentation: Services for stimulant users, *Conference of the Federation of Drug and Alcohol Professionals*. London, 8th November 2006.

Bloor, R., Crome, I., Moss, J., Freeman, S., Okolo, D., & Astari, D. (2006). The Impact of Treatment on Female Drug Using Sex Workers, *NTA Research Briefings*. London: NHS, National Treatment Agency for Substance Misuse.

Bryk, A.S., & Raudenbush, S.W. (2002). *Hierarchical linear models: applications and data analysis methods. Second edition* London: Sage

Chambers, R. (1994). The origins and practice of participatory rural appraisal. *World Development*, 22(7), 953-969.

DeLeon, G., & Jainchill, N. (1986). Circumstances, motivation, readiness and suitability. *Journal of Psychoactive Drugs*, 18, 203-208.

Department of Health, Scottish Office, Department of Health Welsh Office, & Department of Health and Social Services Northern Ireland (1999). *Drug Misuse and Dependence – Guidelines on Clinical Management* London: Department of Health

Donmall, M., Watson, A., Millar, T., & Dunn, G. (2005). *Outcome of waiting lists (OWL) study. Waiting times for drug treatment: effects on uptake and immediate outcomes* London: NTA

Edmunds, M., Dennis, D., Turnbull, P., & Hough, M. (unpublished). *From Pillar to Post: A Study of Non-Engagement in Arrest Referral and Treatment Services*, London: CPRU, South Bank University.

Fielding, N.G., & Fielding, J.L. (1986). *Linking Data* London: Sage

Fiorentine, R., Nakashima, J., & Anglin, M.D. (1999). Client engagement with drug treatment. *Journal of Substance Abuse Treatment*, 17(3), 199-206.

Fischer, J., Jenkins, N., Bloor, M., Neale, J., & Berney, L. (2007). *Drug User Involvement in Treatment Decisions*. York: Joseph Rowntree Foundation, University of Glasgow.

Gilbert, L. (1997). Why Women do and do not Access Street-Based Services. *Drugs News*, 17, 30-34.

Godfrey, C., Stewart, D., & Gossop, M. (2004). Economic analysis of costs and consequences of the treatment of drug misuse: 2-year outcome data from the National Treatment Outcome Research Study (NTORS). *Addiction*, 99(6), 697-707.

Greater London Alcohol and Drug Alliance (2003). *London: The highs and the lows* London: Greater London Authority

Guest, G., Bunce, A., & Johnson, L. (2006). How Many Interviews Are Enough?: An Experiment with Data Saturation and Variability. *Field Methods*, 18(1), 59-82.

Harocopos, A., Dennis, D., Turnbull, P.J., Parsons, J., & Hough, M. (2003). *On the Rocks: A Follow-up Study of Crack Users in London* London: CPRU, South Bank University

Hay, G., Gannon, M., MacDougall, J., Millar, T., Eastwood, C., McKeganey, N., & Gordon, L. (2006). Local and national estimates of the prevalence of opiate use and/or crack cocaine use (2004/05). In N. Singleton,R. Murray, & L. Tinsley (Eds.), *Measuring different aspects of problem drug use: Methodological developments*. London: Home Office.

Hettema, J., Steele, J., & Miller, W.R. (2005). A Meta-Analysis of Research on Motivational Interviewing Treatment Effectiveness (MARMITE). *Annual Review of Clinical Pscychology*, 1, 91-111.

Hiller, M.L., Knight, K., Broome, K.M., & Simpson, D.D. (1998). Legal pressure and treatment retention in a national sample of long-term residential programs. *Criminal Justice and Behaviour*, 25(4), 463-481.

Hsieh, F.Y., Bloch, D.A., & Larsen, M.D. (1998). A simple method of sample size calculation for linear and logistic regression. *Statistics in Medicine*, 17(14), 1623-1634.

Hucklesby, A., Eastwood, C., Seddon, T., & Spriggs, A. (2007). The evaluation of the Restriction on Bail pilots: Final report. Home Office Online Report 06/07. Home Office: London.

Hunter, G., & Powis, B. (1996). *Women Drug Users : Barriers to service use, and service needs*. The Centre for Research on Drugs and Health Behaviour: .

Information and Statistics Division (2004). *An evaluation of why individuals present to and drop out of drug treatment services. Final Report*. Edinburgh: NHS Scotland

Joe, G.W., Simpson, D.D., & Broome, K.M. (1998). Effects of readiness for drug abuse treatment on client retention and assessment of process. *Addiction*, 93(8), 1177-1190.

Joe, G.W., Simpson, D.D., & Broome, K.M. (1999). Retention and patient engagement models for different treatment modalities in DATOS. *Drug and Alcohol Dependence*, 57(2), 113-125.

Layder, D. (1998). *Sociological Practice: Linking Theory and Social Research* London: Sage

Maglione, M., Chao, B., & Anglin, M.D. (2000). Correlates of outpatient drug treatment drop-out among methamphetamine users. *Journal of Psychoactive Drugs*, 32(2), 221-228.

Marsh, J.C., D'Anino, T.A., & Smith, B.D. (2000). Increasing access and providing social services to improve drug abuse treatment for women and children. *Addiction*, 95(8), 1237-1248.

Meier, P. (2005). *A national survey of retention in rehabilitation services* London: NTA

Millar, T., Donmall, M., & Jones, A. (2004). *Treatment Effectiveness: Demonstration analysis of treatment surveillance data about treatment completion and retention.* London: NTA

Miller, W.R., Rollnick, S., & Conforti, K. (2002). *Motivational Interviewing: Preparing People for Change (Second Edition)* New York: Guilford Press

National Audit Office (2004). *Drug Misuse 2004. Reducing the Local Impact* London: NAO

NICE (2007). Psychosocial management of drug misuse. Draft for consultation. London: National Institute for Health and Clinical Excellence.

NTA (2002). *Drug services' briefing: Treating cocaine/crack dependence* London: NTA

NTA (2004). Methadone Series: Engaging and Retaining Clients in Drug Treatment. London: National Treatment Agency for Substance Misuse.

NTA (2005). *Retaining Clients in Drug Treatment. A Guide for Providers and Commissioners* London: National Treatment Agency

NTA (2006). *Performance Information.* London: National Treatment Agency for Substance Misuse.

Peters, R.H., Haas, A.L., & Murrin, M.R. (1999). Predictors of retention and arrest in drug courts. *National Drug Court Review*, 2(1), 33-60.

Petry, N.M. (1999). Therapeutic alliance and psychiatric severity as predictors of completion of treatment for opiod dependence. *Psychiatric Services*, 50(2), 219-227.

Powis, B. (1995). Gender Issues and Treatment Provision:are Equal Numbers Enough? *Addiction Counselling World.*

Ritter, A.J., Fry, C.L., & Swan, A. (2003). The ethics of reimbursing injecting drug users for public health research interviews: What price are we prepared to pay? *International Journal of Drug Policy*, 14(1), 1-3.

Sayre, S.L., Schmitz, J.M., Stotts, A.L., Averill, P.M., Rhoades, H.M., & Grabowski, J.J. (2002). Determining predictors of attrition in an outpatient substance abuse program. *American Journal of Drug and Alcohol Abuse*, 28(1), 55-72.

Seddon, T. (2005). Paying drug users to take part in research: Justice, human rights and business perspectives on the use of incentive payments. *Addiction Research & Theory*, 13(2), 101-109.

Simpson, D.D., Joe, G.W., Rowan-Szal, G.A., & Greener, J.M. (1997). Drug abuse treatment process components that improve retention. *Journal of Substance Abuse Treatment*, 14(6), 565-572.

Stevens, A., Berto, D., Frick, U., Hunt, N., Kerschl, V., McSweeney, T., Oeuvray, K., Puppo, I., Santa Maria, A., Schaaf, S., Trinkl, B., Uchtenhagen, A., & Werdenich, W. (2006). The relationship between legal status, perceived pressure and motivation in treatment for drug dependence: Results from a European study of quasi-compulsory treatment *European Addiction Research*, 12, 197-209.

Strang, J., Best, D., Ridge, G., & Gossop, M. (2004). *Randomised clinical trial of the effects of time on a waiting list on clinical outcomes in opiate users awaiting out-patient treatment* London: NTA

Strang, J., Sheridan, J., Hunt, C., Kerr, B., Gerada, C., & Pringle, M. (2005). The prescribing of methadone and other opioids to addicts: national survey of GPs in England and Wales. *British Journal of General Practice*, 55(515), 444-451.

Swift, W., Copeland, J., & Hall, W. (1996). Characteristics of Women with Alcohol and other Drug Problems: Findings of an Australian National Survey. *Addiction*, 91(8), 1141-1150.

Tabachnick, B.G., & Fidell, L.S. (2001). *Using Multivariate Statistics. 4th Edition.* Needham Heights, MA: Allyn & Bacon

Taylor, D. (2000). The Ecstasy and the Agony, *The Guardian* (p. 6).

Zador, D., & Farrell, M. (2006). Methadone tolerance testing in drug misusers: Beyond the edge of safety. *British Medical Journal*, 333, 1172-1173.
