# Supplementary material for: Early exit: Estimating and explaining early exit from drug treatment
Source: Harm Reduct J. 2008 Apr 25;5:13. doi: 10.1186/1477-7517-5-13 (PMC2391146; doi:10.1186/1477-7517-5-13)
Supplement: Additional file 2 — Interview guide and information sheet. [file 1477-7517-5-13-S2.pdf]

Early Exit

INTERVIEW GUIDE - FINAL

This interview guide lists the subjects to be discussed in interviews with people who have dropped out of drug services. This is a draft that has been developed in consultation between project researchers and an advocate from The Alliance.

The guide is intended to provide starting points for discussion, but respondents should be allowed to structure their responses in their own way. The researcher will therefore use the guide flexibly as an *aide memoire* to ensure that all the relevant topics are covered.

It assumes that information on the interviewee's age, gender and the service they used has already been collected at the time of interview.

TOPIC: Contacting the service

PROMPTS:

When did you start using the service?

How did you get in contact with the service?

In general, what did you think of drug services before you came?

Previous treatment experience
- No of episodes
- Range of modalities
- Range of service providers
- How did this affect expectations and objectives this time? (where applicable)

What did you know about the service before you first met them?

To what extent did you feel under pressure to use the services from other people rather than it being your own choice?

What happened when you first got in touch with the service?

Did you have to wait to start getting the service?

What did you hope to get out of using the service?
- Was it crisis management or long term changes

TOPIC: First impressions

PROMPTS:

What did you think of the service when you first met them?

How were you treated the first time you met someone from the service?

What did you think of the assessment interview?

Did they offer you the service that you wanted?

What did you think of the building where they offered you treatment?

TOPIC: Engaging in the service

PROMPTS:

How many times did you use the service?

What sort of service were you given?

What things about the service, if any, led you to stop seeing them?

Do you feel that the service treated you fairly?

TOPIC: Leaving the service

PROMPTS:

When was the last time you saw the service?

What happened the last time you saw them?

Why did you stop seeing the service?

- You felt your needs had been met
- Motivation to tackle problem use i.e. did they want to stop using.
- Suspicions about confidentiality (especially if referred via CJS)
- Concerns about stigmatisation
- Perceived pressure from drug-using friends or other
- Lifestyle/outside influences
- Concerns about child care issues
- Issues related to choice of treatment options/ opening times/
- Whether it was convenient to attend etc
- Issues connected with allied services/organisations e.g. pharmacies

Have you spoken to anyone else about the service? If so, what did you tell them?

Would you consider having further treatment (at any service) in the future?

If so, would you be likely to go back to the service you recently left?

TOPIC: Outcomes

PROMPTS:

What do you think you got out of using the service?

What's happened with your drug use since you first contacted the the service?

TOPIC: Barriers

PROMPTS:

Is there anything that you can think of that would stop people using the service if they had problems with drugs?

What stopped you from going back to the service?

What would make it easier for people to use the service?

TOPIC: Improvements

How do you think the service could have helped you more?

How could the service be improved for other people?

If there were one change that could be made to the service which would have made it more likely for you to stay in treatment, what would it be?

END

INFORMATION SHEET

Early Exit Research Project

This information is about the Early Exit research project. It is for people who have recently left drug treatment.

You are being invited to take part in a research study. Before you decide, it is important for you to understand why the research is being done and what it will involve. Please take time to read the following information carefully and discuss it with others if you wish. Ask us if there is anything that is not clear or if you would like more information. You can take time to decide whether or not you wish to take part.

Thank you for reading this.

The research is to find out how to improve drug treatment services. It will last until the end of November 2006.

We have chosen you from the people who have recently left drug treatment. We chose you because we believe that you will have something interesting to tell us.

It is up to you to decide whether or not to take part. If you do decide to take part you will be given this information sheet to keep and you will sign two consent forms (you can keep one of them). If you decide to take part, you are still free to withdraw at any time and without giving a reason.

If you decide to take part, we will interview you once. We will pay you £15 for this interview.

During the interview, we will ask you to tell us about your health, your drug and alcohol use, and your experience of drug treatment.

If you take part in the research, this will have no effect on any treatment you receive in future. We hope that the research will be used to improve drug treatment.

If you have any complaints about this research project, you can complain about it through the complaints procedure of Treatment Service.

The research is confidential. We will not tell anyone else what we talk about. We will only pass on information to the appropriate authorities if you tell us that you intend to harm yourself or another named person.

We would like your permission to ask information about you from Treatment Service.

If you take part in the research, we will send you a summary of the results.

This research is funded by the Department of Health. It has been ethically reviewed by the South-East Multi-Regional Ethics Committee of the NHS. It is being carried out by the University of Kent.

If you want more information about the study you can:

- Ask the person who gave you this information;
- Ask your keyworker at Name of Drug Treatment Service;
- Contact Polly Radcliffe or Alex Stevens at the University of Kent (Tel – 01227 827 304 – direct line).

They can phone you straight back if you are calling from a phone that takes incoming calls. Just ask. But they are not in the office all the time. Be prepared to leave details of how they can contact you or when you will call again.

Thanks.
